# Exercise and physical activity for fatigue, disease activity and immune-inflammatory outcomes in adults with systemic lupus erythematosus: an umbrella review

**DOI:** 10.3389/fimmu.2026.1925582

**Published:** 2026-09-18

**Authors:** Jingwei Fang, Xiaoge Ma, Mohan Sun, Shuwei Chen, Zhengda Ma, Weibao Liang

**Affiliations:** 1 Faculty of Health Sciences and Sports, Macao Polytechnic University, Macao, China; 2 School of Physical Education and Leisure, Guangdong Ocean University, Zhanjiang, China; 3 School of Physical Education, Xi’an Peihua University, Xian, China; 4 Institute of Athletic Training Science, Capital University of Physical Education and Sports, Beijing, China; 5 Hebei Sport University, Shijiazhuang, China; 6 School of Physical Education and Sports Science, South China Normal University, Guangzhou, China

**Keywords:** disease activity, exercise, fatigue, inflammation, physical activity, sedentary behavior, systemic lupus erythematosus, umbrella review

## Abstract

**Background:**

Fatigue and impaired health-related quality of life are common in systemic lupus erythematosus (SLE), and persistent immune-inflammatory activation may contribute to symptom burden even when clinical disease activity is controlled. Exercise and physical activity are recommended as non-pharmacological adjuncts, but existing systematic reviews overlap and report discordant conclusions. We undertook an umbrella review to synthesize evidence on clinical outcomes and to map immune-inflammatory outcomes.

**Methods:**

We searched PubMed, Embase, the Cochrane Library, Web of Science, Scopus and SPORTDiscus from inception to 1 March 2026 for systematic reviews, with or without meta-analysis, of exercise, physical activity, sedentary behavior or related lifestyle/self-management interventions in adults with SLE. Methodological quality was assessed with AMSTAR-2, overlap with the corrected covered area (CCA), and certainty with GRADE for intervention comparisons and the Ioannidis classification for observational associations. Because primary studies overlapped substantially, review estimates were not pooled; findings were synthesized narratively by construct and comparator, prioritizing Core-eligible reviews.

**Results:**

Eighteen reviews were included; seven were Core-eligible. AMSTAR-2 confidence was high in one review, low in five and critically low in twelve, and overall overlap was very high (CCA 19.6%). For fatigue versus usual care, two Core-eligible reviews reported statistically significant reductions (MD −0.61, 95% CI −1.19 to −0.02; 2 trials, 115 participants; and MD −0.52, 95% CI −0.91 to −0.13; 3 trials, 135 participants), whereas the high-confidence Cochrane review found no significant difference (MD −0.59, 95% CI −1.40 to 0.22; 2 trials, 104 participants); certainty was low, and all three estimates derived from an overlapping pool of four small primary trials. Disease-activity estimates were not statistically significant. Withdrawal comparisons were statistically inconclusive and of very low certainty; flare evidence was sparse and ungraded.

**Conclusions:**

Exercise and physical activity may slightly reduce fatigue in adults with SLE, but the evidence is low-certainty and substantially overlapping. Current review-level evidence does not establish a robust immune-inflammatory mechanism or a disease-activity effect, and safety and tolerability remain uncertain. Future trials should prespecify fatigue, disease activity, flares and immunological endpoints.

**Systematic Review Registration:**

https://www.crd.york.ac.uk/PROSPERO/view/CRD420261392698, identifier CRD420261392698.

## Introduction

Systemic lupus erythematosus (SLE) is a chronic, multisystem autoimmune disease that predominantly affects women of reproductive age and follows a relapsing–remitting course. Beyond organ involvement, most patients experience fatigue, reduced physical fitness, pain, mood disturbance and impaired health-related quality of life, and these symptoms frequently persist even when serological and clinical disease activity is well controlled. Fatigue in particular is among the most prevalent and disabling features of the disease and is consistently rated by patients as a priority for management (1, 2).

Because pharmacological therapy alone does not fully address this symptom burden, non-pharmacological strategies have become an increasingly important component of care. Recent European Alliance of Associations for Rheumatology (EULAR) recommendations for the non-pharmacological management of SLE were informed by systematic literature reviews of exercise, physical activity and other lifestyle interventions (3–5). The rationale for exercise is biologically and clinically plausible: people with SLE are frequently deconditioned and physically inactive, which may aggravate fatigue, cardiovascular risk and functional limitation, and structured activity has the potential to interrupt this cycle without adding pharmacological toxicity.

Over the past decade a substantial number of systematic reviews, several with meta-analyses, have evaluated exercise and physical activity in SLE (6–8). However, these reviews differ markedly in their scope — some focus on structured aerobic or resistance exercise, others on broader physical activity, sedentary behavior, or multicomponent lifestyle and self-management interventions — and they employ heterogeneous outcome measures, comparators and analytical methods. Critically, many of them draw on the same small pool of primary trials, so their conclusions are not independent, and they vary considerably in methodological rigor. As a result, the literature is fragmented and at times discordant, which makes it difficult for clinicians, guideline developers and patients to judge the overall strength and direction of the evidence.

An umbrella review (overview of reviews) is well suited to this situation, because it synthesizes and critically appraises the existing body of systematic reviews rather than individual studies, quantifies the overlap among them, grades the certainty of the pooled evidence, and identifies gaps. We therefore conducted an umbrella review to summarize and appraise systematic reviews of exercise, physical activity, sedentary behavior and related lifestyle and self-management interventions in adults with SLE, with the aims of (i) characterizing the available reviews and their methodological quality, (ii) quantifying the degree to which they overlap in primary studies, (iii) summarizing the direction and certainty of the evidence for fatigue, disease activity, tolerability and related outcomes, and (iv) clarifying where the evidence is strongest and where it remains insufficient.

## Methods

### Design, protocol and reporting

This was an umbrella review of systematic reviews. The methods were specified in advance and the review was registered with the International Prospective Register of Systematic Reviews (PROSPERO; registration CRD420261392698). The conduct of the review followed established guidance for umbrella reviews and overviews of reviews (9, 10), and reporting follows the Preferred Reporting Items for Systematic Reviews and Meta-Analyses (PRISMA) 2020 statement together with the PRIOR (Preferred Reporting Items for Overviews of Reviews) reporting guideline (11, 12). The literature search is reported in accordance with the PRISMA-S extension (13). After protocol registration, we added two descriptive, non-pooled robustness analyses—an AMSTAR-2 confidence-restricted synthesis and a complete-containment analysis—to clarify methodological confidence and evidence-base nesting; neither addition changed the included review set or any reported effect estimate.

### Eligibility criteria

Reviews were eligible if they met the following criteria. Population: adults (aged ≥18 years) with a diagnosis of SLE; reviews of broader populations (for example, rheumatic and musculoskeletal diseases or idiopathic inflammatory myopathies) were eligible when SLE-specific or exercise-specific data could be identified. Intervention or exposure: structured exercise, physical activity, sedentary behavior, or related lifestyle and self-management interventions, whether evaluated as interventions or as exposures. Comparators: usual care, no intervention, an active or alternative comparator, or a correlational reference group. Outcomes: fatigue, disease activity, health-related quality of life, physical fitness or aerobic capacity, inflammatory or immune markers, and safety outcomes including disease flares and withdrawals. Study design: systematic reviews, with or without meta-analysis. To keep the primary synthesis focused on the most directly relevant evidence, each review was classified as “Core-eligible” if it fully met the prespecified population, intervention, comparator, outcome and design criteria, or as contributing to the “evidence map” if it met them only partially (for example, a broader-population review with an identifiable SLE exercise subset). Core-eligible reviews informed the primary conclusions; evidence-map reviews were used for context.

### Information sources and search

We searched six bibliographic databases — PubMed, Embase, the Cochrane Library, Web of Science, Scopus and SPORTDiscus — from inception to 1 March 2026, with no language restriction applied at the search stage. The search combined controlled vocabulary and free-text terms for SLE, for exercise, physical activity and sedentary behavior, and for systematic review or meta-analysis publication types. The complete, executable search strategy for each database is provided in **Supplementary Data Sheet 1**: **Supplementary Table 1**. The number of records retrieved from each database in the dated 1 March 2026 export is reported alongside the corresponding search string in **Supplementary Table 1** and in **Figure 1**. For the Cochrane Library, only records in the Cochrane Database of Systematic Reviews were counted and screened, because records in the Central Register of Controlled Trials are reports of primary trials and are ineligible under the study-design criterion.

**Figure 1:**
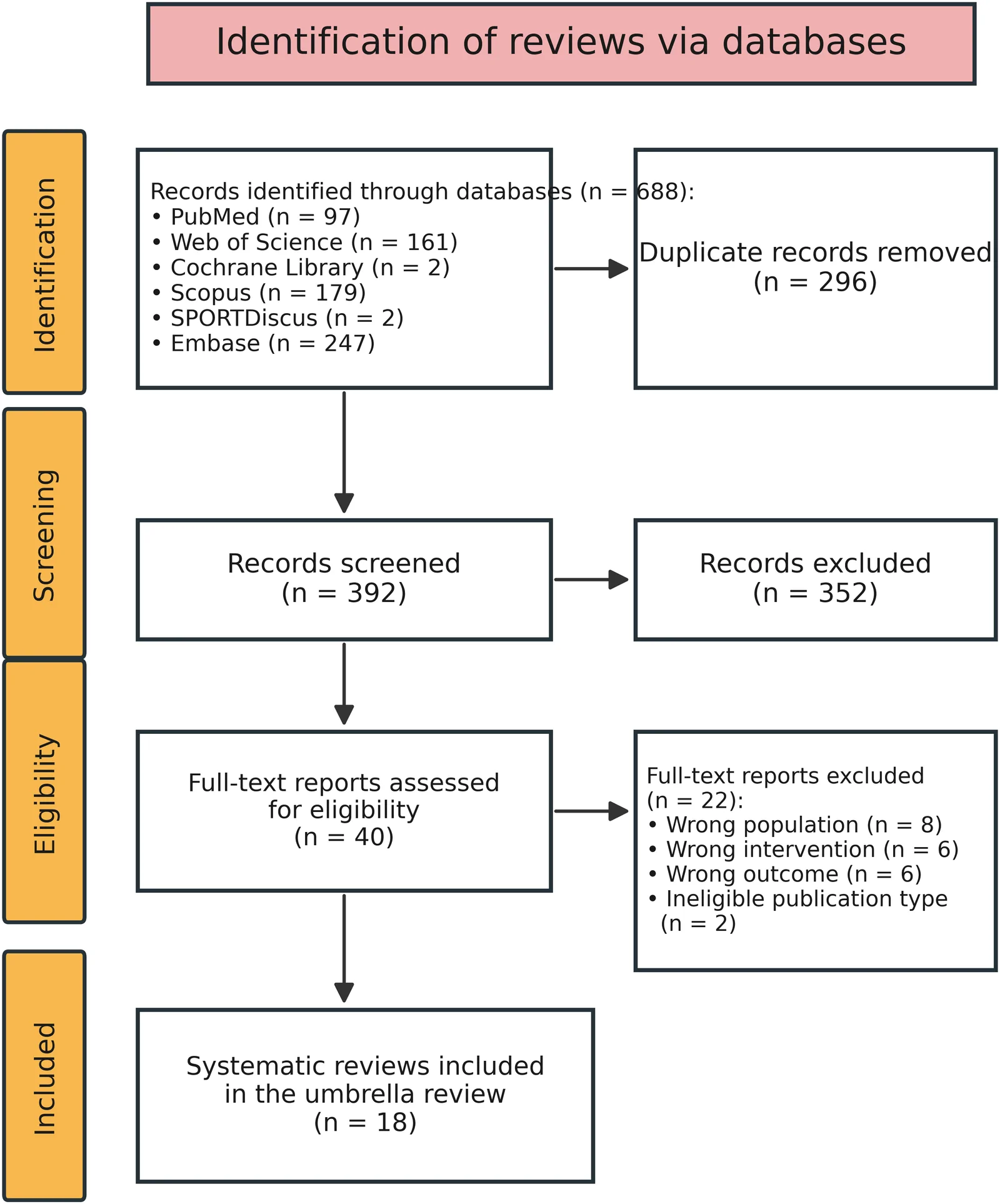
PRISMA 2020 flow diagram of study selection. Flow of records through identification, screening and inclusion for this umbrella review, following the PRISMA 2020 statement. Record counts are shown within the diagram. Database-level record counts are also reported alongside each search string in **Supplementary Data Sheet 1**: **Supplementary Table 1**. PRISMA, Preferred Reporting Items for Systematic Reviews and Meta-Analyses; SLE, systemic lupus erythematosus.

### Study selection and data extraction

Titles and abstracts and then full texts were screened independently by two reviewers against the eligibility criteria. Data were extracted independently by two reviewers into piloted structured forms capturing review characteristics (authors, year, design, number and type of included primary studies), population, intervention or exposure, comparators, outcomes, reported effect estimates with confidence intervals, heterogeneity statistics and the number of contributing primary studies, the risk-of-bias tool used, and any certainty rating reported by the review. Disagreements at either stage were resolved through discussion, with adjudication by a third reviewer when consensus could not be reached. The full extracted outcome-level evidence is provided in **Supplementary Data Sheet 1**: **Supplementary Table 2**. For every pooled estimate reported by a Core-eligible review, we additionally recorded from the source review the individual primary studies contributing to that estimate, their study design as labelled by the source review, the number of participants analyzed in each arm, the comparator and the outcome instrument (**Supplementary Table 6**). Before resubmission, all full-text eligibility decisions, outcome-level extractions and record counts were audited against the source publications and dated database exports; this audit did not change the set of included reviews or any reported effect estimate.

### Methodological quality of included reviews

The methodological quality of each review was appraised with AMSTAR-2 (A MeaSurement Tool to Assess systematic Reviews, version 2) (14). Overall confidence in the results of a review was rated as high (no or one non-critical weakness), moderate (more than one non-critical weakness but no critical weakness), low (one critical weakness, with or without non-critical weaknesses) or critically low (more than one critical weakness); the critical domains are items 2, 4, 7, 9, 11, 13 and 15. Item-level assessments are presented in **Supplementary Data Sheet 1**: **Supplementary Table 3** and summarized graphically in **Figure 2**.

**Figure 2:**
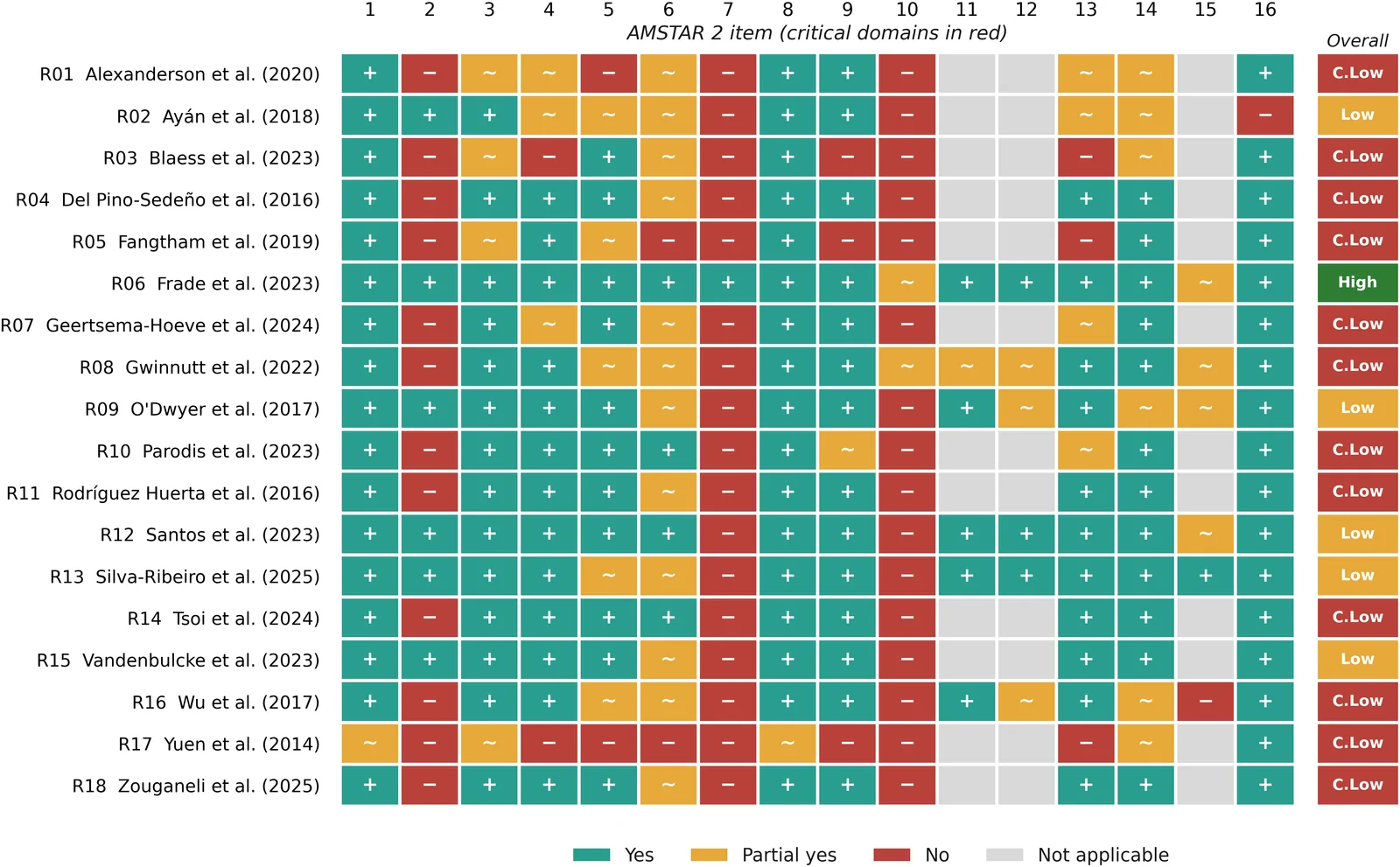
Methodological quality of included systematic reviews assessed with AMSTAR 2. Item-level AMSTAR 2 assessments for included reviews of exercise and physical activity in systemic lupus erythematosus. Each cell shows the rating for one item (green, yes; amber, partial yes; red, no; grey, not applicable); the right-hand column gives the overall confidence in the results of the review. Full item definitions are given in **Supplementary Data Sheet 1**: **Supplementary Table 3**. AMSTAR 2, A MeaSurement Tool to Assess systematic Reviews, version 2.

### Overlap among reviews

Because systematic reviews of the same field commonly include the same primary studies, we quantified overlap using the corrected covered area (CCA) (15), calculated overall and within each prespecified intervention or exposure cluster. CCA values were interpreted conventionally as slight (0–5%), moderate (6–10%), high (11–15%) or very high (>15%) overlap. The full pairwise overlap matrix is shown in **Figure 3**. We further examined whether the set of primary studies included in one review was entirely contained within that of another (complete containment, or nesting), because a review whose evidence base is fully encapsulated by a larger review contributes no independent primary data; the resulting hierarchy is reported in **Supplementary Table 8** and **Supplementary Figure 1**.

**Figure 3:**
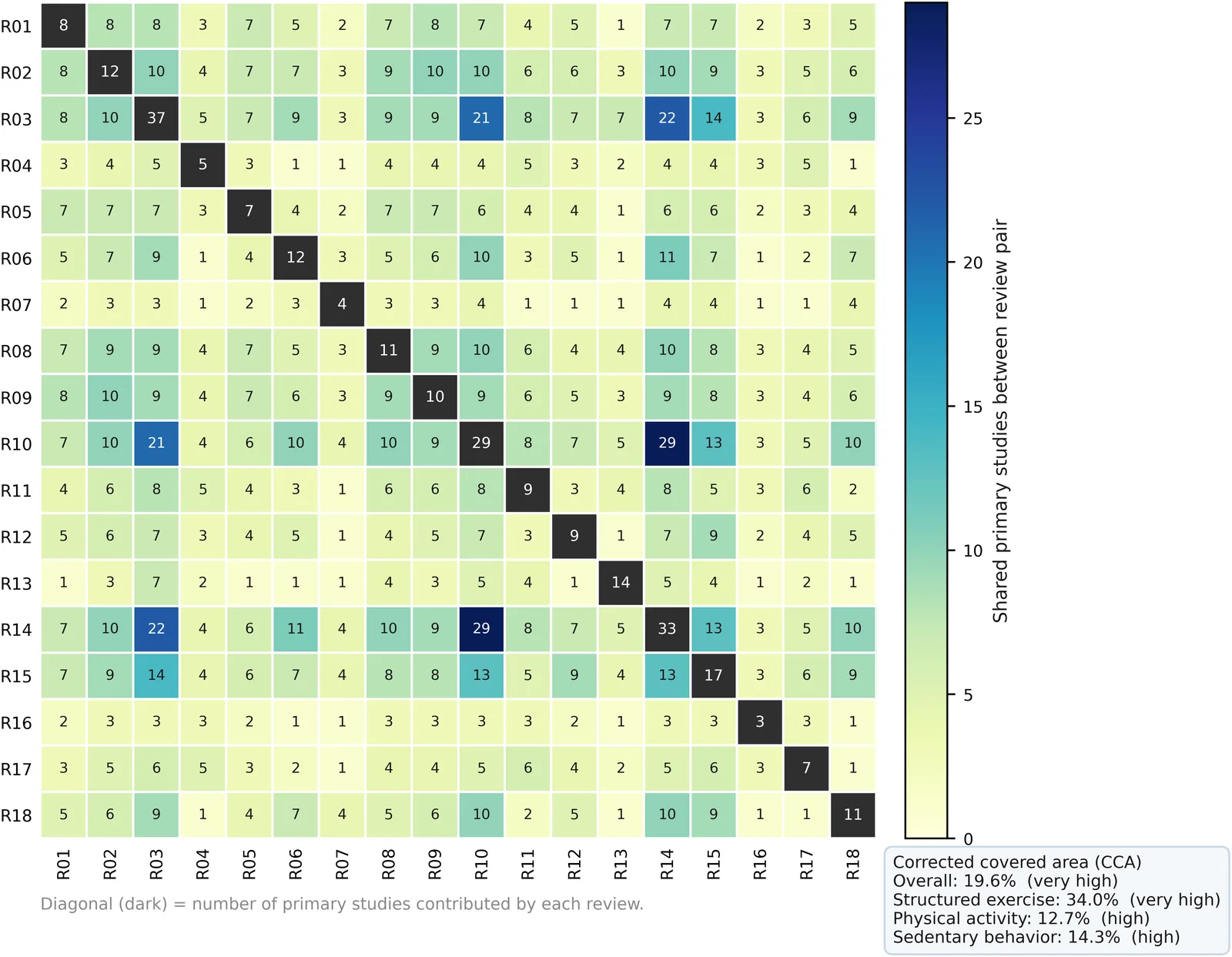
Primary-study overlap among included reviews of exercise and physical activity in SLE. Pairwise counts of primary studies shared by each pair of reviews. Cell shading indicates shared study counts; the dark diagonal shows the number of primary studies contributed by each review. CCA values for each prespecified intervention/exposure cluster and overall are shown in the annotation box and in **Supplementary Data Sheet 1**: **Supplementary Table 5**. Because overlap was high to very high, review estimates are presented separately rather than pooled. Complete containment (nesting) of primary-study sets between reviews, which the pairwise matrix does not display, is shown separately in **Supplementary Figure 1** and **Supplementary Data Sheet 1**: **Supplementary Table 8**. CCA, corrected covered area; R01–R18, review identifiers as listed in **Table 1**; SLE, systemic lupus erythematosus.

### Certainty of evidence and synthesis

Certainty of evidence was assessed with the Grading of Recommendations Assessment, Development and Evaluation (GRADE) approach for intervention comparisons (28); where a Core-eligible review reported its own GRADE rating, that rating was retained. When a Core-eligible review did not provide a GRADE rating, two reviewers independently applied GRADE to the review-level estimate, starting randomized intervention evidence at high certainty and considering risk of bias, inconsistency, indirectness, imprecision and publication bias; disagreements were resolved by consensus. For observational exposure–outcome associations, the Ioannidis hierarchy was applied provisionally. Because the source review did not report all inputs required to verify Classes I–III, statistically significant associations were classified as provisional Class IV, the weakest category of evidence. Ratings were summarized at the level of the clinical construct and comparator; where contributing reviews differed, the most cautious rating is reported. Review-level certainty details and domain-level rationales are provided in **Supplementary Data Sheet 1**: **Supplementary Table 4**.

Because the included reviews shared many of the same primary studies (high to very high CCA) and reported heterogeneous metrics and comparators, their estimates were not statistically combined across reviews; pooling would have counted the same trials more than once. Instead, the evidence was synthesized narratively and organized by clinical construct (fatigue, disease activity, tolerability and other outcomes) and by comparator, presenting each review’s estimate separately and giving primacy to Core-eligible reviews. Intervention evidence and observational exposure associations were kept separate throughout.

### Sensitivity synthesis by methodological confidence

Core eligibility was defined by direct relevance to the prespecified population, intervention, comparator, outcome and design criteria, and not by methodological quality; relevance and quality are distinct properties, and a review can address the target question directly while remaining methodologically weak. To keep the two separable, evidence tier was cross-tabulated against AMSTAR-2 overall confidence and the construct-level synthesis was repeated in two restricted sets: first, Core-eligible reviews rated high or low overall confidence, excluding those rated critically low; and second, the single review rated high confidence. These restricted syntheses were descriptive and involved no re-pooling of estimates. They are reported in **Supplementary Table 7**.

## Results

### Search results and selection

The database searches identified 688 records (PubMed, n=97; Embase, n=247; Scopus, n=179; Web of Science, n=161; Cochrane Library, n=2; SPORTDiscus, n=2). After 296 duplicate records were removed, 392 records were screened, of which 352 were excluded at title and abstract. Of 40 full-text reports assessed, 22 were excluded (wrong population, n=8; wrong intervention, n=6; wrong outcome, n=6; ineligible publication type, n=2). Eighteen systematic reviews met the inclusion criteria and were included in the umbrella review. The flow of records is shown in **Figure 1**. Database-level counts are also given alongside each search string in **Supplementary Table 1**. The principal clinical estimates are displayed in **Figure 4**, methodological quality in **Figure 2**, review overlap in **Figure 3**, and construct-level certainty in **Figure 5**.

**Figure 4:**
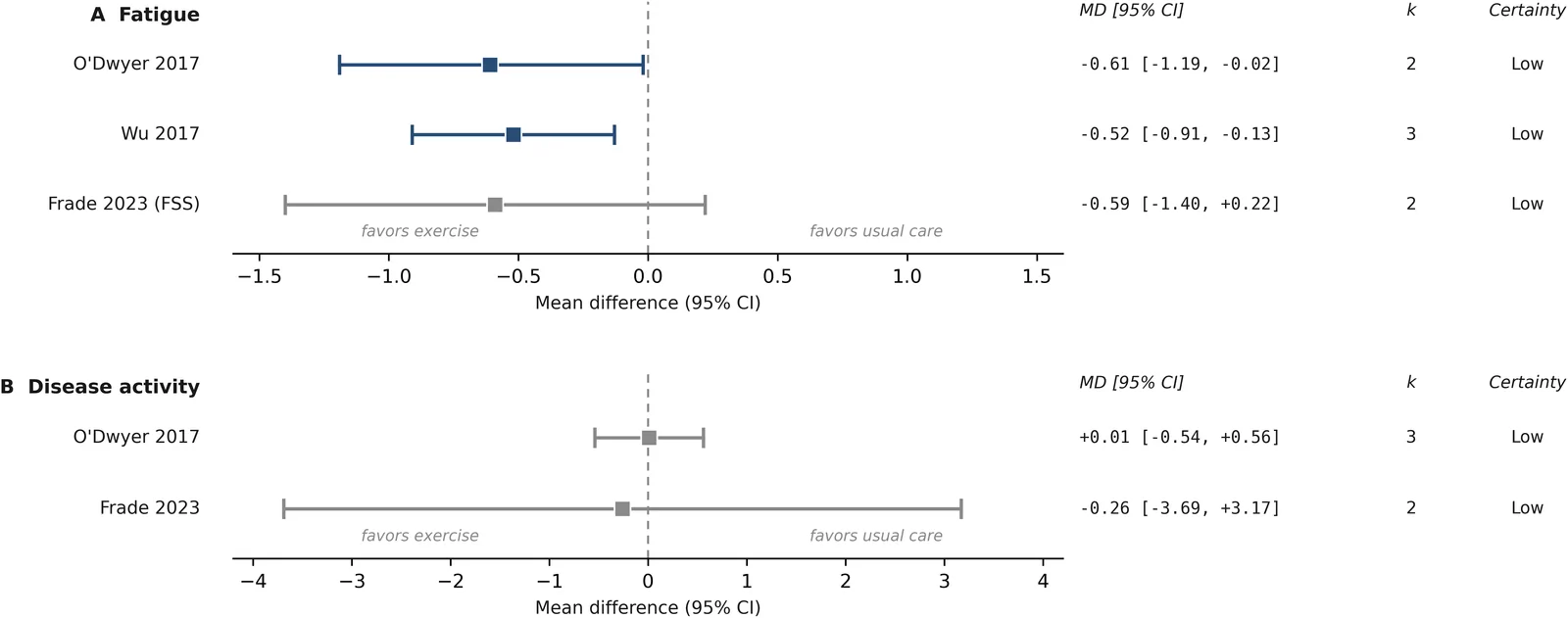
Forest plot of fatigue and disease activity, exercise versus usual care (mean difference). **(A)**, fatigue; **(B)**, disease activity. Each row shows a single Core-eligible review’s pooled mean difference (square) with its 95% confidence interval (horizontal line) for exercise versus usual or standard care; the number of contributing primary studies (k) and the certainty of evidence are shown to the right. A negative mean difference favors exercise (lower fatigue and lower disease-activity scores). Estimates are presented on their original scales and are not combined across reviews, because the reviews share many of the same primary studies (see **Figure 3**); no summary diamond is therefore shown. Standardized differences and other comparators are summarized in **Table 3**. The three fatigue estimates in panel A derive from an overlapping pool of four primary trials (29–32), representing 174 participants in the relevant analyzed arms when each trial is counted once; the two disease-activity estimates in panel B derive from five trials. The rows are therefore not independent replications of the same comparison (**Supplementary Data Sheet 1**: **Supplementary Table 6**). CI, confidence interval; FSS, Fatigue Severity Scale; k, number of primary studies; MD, mean difference.

**Figure 5:**
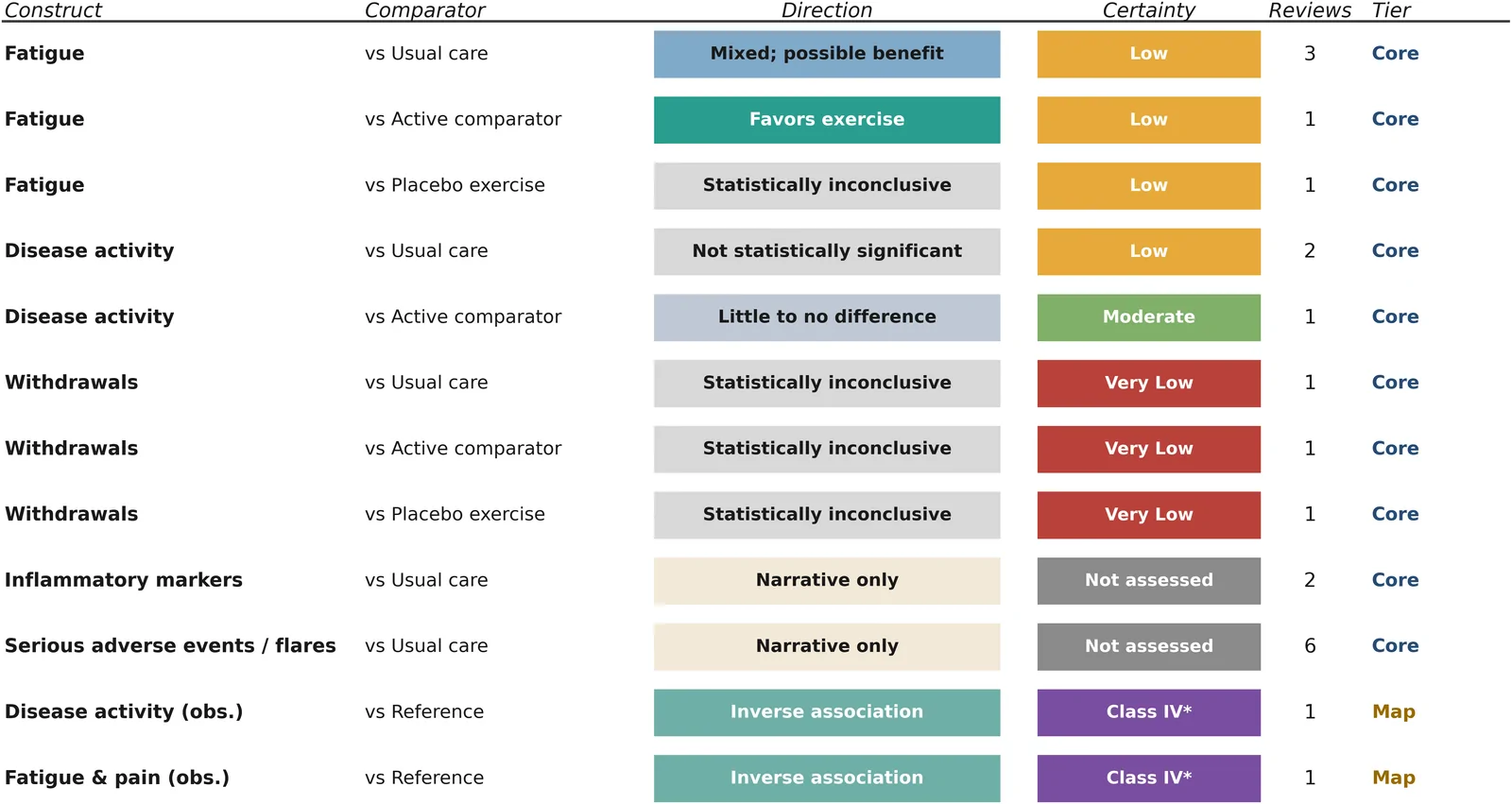
Certainty and direction of evidence for clinical and immune-inflammatory outcomes in SLE. Summary of the direction and certainty of evidence for each construct and comparator. Direction tiles distinguish mixed or potentially beneficial findings, statistically inconclusive estimates, little-to-no-difference estimates, inverse observational associations and narrative-only evidence; they do not treat non-significance as proof of equivalence. Certainty badges show the GRADE rating or, for observational exposure associations, a provisional Ioannidis class. The number of contributing reviews and evidence tier are shown to the right. Ratings are summarized at the construct level (see **Table 4**; **Supplementary Data Sheet 1**: **Supplementary Table 4**). “Class IV*” denotes a provisional Class IV classification because the source review did not report all inputs required to verify Classes I–III. Intervention and observational evidence are kept separate. GRADE, Grading of Recommendations Assessment, Development and Evaluation; obs., observational; SLE, systemic lupus erythematosus.

### Characteristics of included reviews

The 18 included reviews were published between 2014 and 2025 and comprised narrative and quantitative systematic reviews, Cochrane and EULAR-commissioned reviews, and recommendation-informing systematic literature reviews (3–8, 16–27). Seven reviews were classified as Core-eligible — Ayán et al. (8), Blaess et al. (17), Frade et al. (7), O’Dwyer et al. (6), Vandenbulcke et al. (24), Wu et al. (25) and Zouganeli et al. (27) — and the remaining eleven contributed to the evidence map (3–5, 16, 18–23, 26). Populations were adults with SLE, although several reviews covered broader rheumatic or inflammatory conditions from which an SLE or exercise subset was identified. Interventions spanned structured aerobic, resistance and combined exercise, broader physical activity, sedentary behavior, and multicomponent lifestyle and self-management programs, and comparators included usual or standard care, control, relaxation or alternative active interventions. The characteristics of each review, including the number of included primary studies, available participant totals and the risk-of-bias tool used, are summarized in **Table 1**. Across the 18 reviews, 55 distinct primary studies were represented once linked reports of the same protocol were collapsed.

**Table 1:** Characteristics of included systematic reviews.

Review	Design	Included studies, n	Population	Intervention/exposure	Comparators	Main outcomes	Primary-study RoB tool	AMSTAR-2	Evidence tier
R01 Alexanderson et al. (16)	Systematic literature review (recommendation-informing)	8 SLE RCTs	IIM and SLE (SLE data only; 312 participants across 8 SLE trials)	Exercise therapy/physical activity	Usual care or non-exercise controls	Fatigue, disease activity, aerobic capacity, inflammatory markers, safety	NR	Critically Low	Evidence map
R02 Ayán et al. (8)	Systematic review	14 (10 RCTs)	People with SLE (aggregate n NR)	Physical exercise	Control, usual care, or alternative exercise	Disease activity, fatigue, physical fitness, adverse effects	PEDro; MINORS	Low	Core-eligible
R03 Blaess et al. (17)	Systematic review	40 (13 RCTs)	Patients with SLE (n=2,291 across included studies)	Physical activity; aerobic and resistance training; cross-sectional exposure	Inactive/lower-activity groups, usual care, baseline	Physical activity, fitness, fatigue, HRQoL, disease activity, cytokines, safety	NR	Critically Low	Core-eligible
R04 Del Pino-Sedeño et al. (18)	Systematic review	12 (7 RCTs); 5 exercise	Adults with SLE (n=549 overall; n=233 in 5 exercise studies)	Non-pharmacologic interventions (exercise subset)	Usual care, relaxation, stretching, or baseline	Fatigue (exercise subset also disease activity, safety)	SIGN	Critically Low	Evidence map
R05 Fangtham et al. (19)	Systematic review without meta-analysis	15 (8 exercise RCTs)	Adults with SLE (n=846 overall; exercise-subset aggregate n NR)	Non-pharmacologic therapies (exercise subset)	Usual care, control, or active non-pharmacologic comparator	Fatigue, depression, pain, HRQoL, disease activity	NR	Critically Low	Evidence map
R06 Frade et al. (7)	Cochrane systematic review and meta-analysis	13 trials (10 RCTs, 3 quasi-RCTs)	Adults with SLE (n=540 commenced treatment; 13 trials)	Structured exercise plus usual pharmacological care	Placebo exercise, usual care alone, or other non-pharmacologic control	Fatigue, functional capacity, disease activity, HRQoL, pain, serious adverse events	Cochrane risk-of-bias tool	High	Core-eligible
R07 Geertsema-Hoeve et al. (20)	Systematic review	25 RCTs	Adults with SLE (n=1,521 overall; n=253 in the relevant physical-activity/QoL subset)	Lifestyle interventions (physical activity category)	Usual care or control	Disease activity, HRQoL; fatigue where reported	SIGN	Critically Low	Evidence map
R08 Gwinnutt et al. (4)	Systematic reviews and meta-analyses (EULAR)	RMD review; SLE exercise subset	RMDs including SLE (SLE exercise-subset aggregate n NR)	Physical exercise and body weight (SLE exercise findings)	Controls or alternative exercise	Disease activity, fatigue, pain, function, HRQoL	Abbreviated Cochrane risk-of-bias tool	Critically Low	Evidence map
R09 O'Dwyer et al. (6)	Systematic review with meta-analyses	11 trials (6 RCTs, 5 quasi-RCTs)	Adults with SLE (n=469)	Exercise and physical activity interventions	Usual care, control, relaxation, or other comparators	Disease activity, fatigue, aerobic capacity, depression, adverse events	Cochrane risk-of-bias tool	Low	Core-eligible
R10 Parodis et al. (3)	Systematic literature review (EULAR)	116 SLE records	SLE and systemic sclerosis (SLE data only; exercise/physical-activity subset aggregate n NR)	Non-pharmacological management (exercise/physical activity subset)	Control or active comparators	Fatigue, disease activity, aerobic capacity, depression, HRQoL	NR	Critically Low	Evidence map
R11 Rodríguez Huerta et al. (21)	Systematic review	21; 9 exercise (4 RCTs)	Adults with SLE (exercise-subset aggregate n NR)	Healthy lifestyle habits (exercise subset)	Control, usual care, relaxation, baseline, or alternative	Fatigue, disease activity/damage, fitness	SIGN	Critically Low	Evidence map
R12 Santos et al. (5)	Systematic review and meta-analysis (EULAR)	55 of 82 RCTs	Inflammatory RMDs including SLE (SLE exercise-subset aggregate n NR)	Non-pharmacological interventions (SLE exercise/physical activity fatigue meta-analysis)	Usual care/control	Fatigue and safety	Cochrane risk-of-bias tool	Low	Evidence map
R13 Silva-Ribeiro et al. (22)	Systematic review and meta-analysis	54 meta-analyzed	Adults with SLE (physical-activity/sedentary-behavior subset aggregate n NR)	Physical activity and sedentary behavior exposure (and other psychosocial factors)	Lower/no exposure, reference group, or correlational comparison	Disease/illness activity, fatigue and pain, adjustment	ROBINS-E/ROBINS-I	Low	Evidence map
R14 Tsoi et al. (23)	Systematic literature review	102; 39 exercise	Patients with SLE (exercise-subset aggregate n NR)	Lifestyle interventions (physical exercise domain)	Usual care/control/baseline/alternative	Fatigue, depression, aerobic capacity, function, disease activity	JBI critical-appraisal checklists	Critically Low	Evidence map
R15 Vandenbulcke et al. (24)	Systematic review	17 (11 RCTs)	Patients with SLE (aggregate n NR)	Physical exercise interventions	Standard care, alternative treatment, different exercise, or observational	Fatigue and safety/disease flare	PEDro; JBI tools	Low	Core-eligible
R16 Wu et al. (25)	Systematic review and meta-analysis	3 (2 RCTs, 1 quasi)	Adults with SLE (n=163)	Aerobic exercise training	Control, usual care, or other comparator	Fatigue and SF-36 vitality	PEDro	Critically Low	Core-eligible
R17 Yuen et al. (26)	Systematic review	7 exercise (4 RCTs)	Patients with SLE (n=160 across 4 RCTs; total exercise-subset n NR)	Fatigue-management strategies (aerobic exercise subset)	Controls or usual care	Fatigue, vitality, disease activity, adverse effects	NR	Critically Low	Evidence map
R18 Zouganeli et al. (27)	Systematic review of RCTs	12 RCTs	Adults with SLE (n=619; plus 8 healthy controls in one trial)	Exercise training (aerobic, resistance, combined, vibration, home-based)	Usual care, control, or alternative exercise	Aerobic capacity, HRQoL, fatigue, depression, pain, disease activity	RoB 2	Critically Low	Core-eligible

Reviews are ordered by first-author surname (R01–R18). Counts give the number of included primary studies (with randomized controlled trials in parentheses); where a review covered a broader population or intervention, the SLE- or exercise-relevant figure is shown. ‘Included studies, n’ therefore refers to the subset informing this umbrella review where applicable. Participant totals in the Population column are reproduced from the source review; the relevant SLE or exercise subset is shown when reported, and NR denotes not reported. These totals should not be summed because primary studies overlap across reviews. Participant counts for individual pooled estimates are given in **Supplementary Data Sheet 1**: **Supplementary Table 6**. Evidence tier: Core-eligible reviews fully met the prespecified population, intervention, comparator, outcome and study-design criteria and inform the primary synthesis; evidence-map reviews met them only partially and are used for context. AMSTAR-2, A MeaSurement Tool to Assess systematic Reviews, version 2; EULAR, European Alliance of Associations for Rheumatology; HRQoL, health-related quality of life; IIM, idiopathic inflammatory myopathies; JBI, Joanna Briggs Institute; MINORS, Methodological Index for Non-Randomized Studies; NR, not reported; PEDro, Physiotherapy Evidence Database scale; RCT, randomized controlled trial; RMD, rheumatic and musculoskeletal disease; RoB, risk of bias; RoB 2, revised Cochrane risk-of-bias tool for randomized trials; ROBINS-E/ROBINS-I, Risk Of Bias In Non-randomized Studies of Exposures/Interventions; SIGN, Scottish Intercollegiate Guidelines Network; SLE, systemic lupus erythematosus.

### Methodological quality

Overall confidence in the results was rated high for one review (the Cochrane review by Frade et al. (7)), low for five reviews and critically low for the remaining twelve (**Table 2**; **Supplementary Data Sheet 1**: **Supplementary Table 3**; **Figure 2**). The most frequent critical weaknesses were the absence of a registered protocol or *a priori* design (item 2) and the absence of a list of excluded studies with justification (item 7); several reviews additionally had limitations in the comprehensiveness of the search (item 4) or in structured risk-of-bias assessment (item 9). The significant fatigue benefits summarized below derive largely from low-confidence reviews, whereas the single high-confidence review reached more conservative conclusions for most comparisons, and the evidence should be read in that light. Evidence tier and methodological confidence were only weakly aligned: of the seven Core-eligible reviews, one was rated high, three low and three critically low. Core eligibility therefore indicates direct relevance to the review question, not methodological quality, and the two properties are reported separately throughout (**Table 2**; **Supplementary Table 7**).

**Table 2:** Methodological quality of included reviews (AMSTAR-2) and summary appraisal.

Review	Overall confidence	Critical domains not satisfied	Summary appraisal	Evidence tier
R01 Alexanderson et al. (16)	Critically Low	2:No a-priori protocol/registration; 7:No excluded-study list	Recommendation-informing SLR with GRADE appraisal, but no registration/protocol and no excluded-study list; selection was not independently duplicated. Search was broad but process limitations create more than one critical weakness.	Evidence map
R02 Ayán et al. (8)	Low	7:No excluded-study list located	PROSPERO registration and PEDro/MINORS appraisal were reported. Search used three databases and no excluded-study list was located; primary-study funding and author conflicts were incompletely reported.	Core-eligible
R03 Blaess et al. (17)	Critically Low	2:No protocol/registration; 4:Limited single-database search; 7:No excluded-study list; 9:No structured RoB appraisal; 13:RoB not integrated	The review reports independent screening, but the search was limited, no protocol or excluded-study list was located, and no structured RoB appraisal was reported.	Core-eligible
R04 Del Pino-Sedeño et al. (18)	Critically Low	2:No protocol/registration; 7:No excluded-study list located	Comprehensive search and SIGN quality appraisal were reported, but no protocol/registration or excluded-study list was located.	Evidence map
R05 Fangtham et al. (19)	Critically Low	2:No protocol/registration; 7:No excluded-study list; 9:RoB appraisal inadequate/unclear	Search and PRISMA flow were reported, but no protocol, no excluded-study list, and one-reviewer/incompletely structured quality appraisal led to multiple critical weaknesses.	Evidence map
R06 Frade et al. (7)	High	None identified	Cochrane review with protocol, comprehensive search, duplicate processes, excluded studies, RoB assessment, appropriate meta-analysis, GRADE SoF tables, and transparent conflicts. Publication-bias assessment was limited by small meta-analyses.	Core-eligible
R07 Geertsema-Hoeve et al. (20)	Critically Low	2:No protocol/registration; 7:No excluded-study list located	Screening and SIGN appraisal were reported, but the authors state no registration/formal protocol; no excluded-study list was located.	Evidence map
R08 Gwinnutt et al. (4)	Critically Low	2:No protocol/registration located; 7:No excluded-study list located	Broad EULAR review with extensive search, **Supplementary Tables**, RoB/AMSTAR2-based evidence grading and outcome synthesis. A protocol/registration and excluded-study list were not located for this review itself.	Evidence map
R09 O'Dwyer et al. (6)	Low	7:No excluded-study list located	Registered systematic review with broad search, duplicate selection, Cochrane RoB and meta-analysis. No excluded-study list was located; publication-bias assessment was constrained by few studies.	Core-eligible
R10 Parodis et al. (3)	Critically Low	2:No protocol/registration located; 7:No excluded-study list located	EULAR SLR with librarian-designed search, duplicate screening/extraction and JBI critical appraisal. No protocol/registration or excluded-study list was located; RoB was performed by one investigator under supervision.	Evidence map
R11 Rodríguez Huerta et al. (21)	Critically Low	2:No protocol/registration located; 7:No excluded-study list located	SIGN appraisal and structured narrative synthesis were reported, but no protocol/registration and no excluded-study list were located.	Evidence map
R12 Santos et al. (5)	Low	7:No excluded-study list located	PROSPERO protocol, comprehensive search, duplicate processes, Cochrane RoB, meta-analysis and Oxford evidence levels were reported. No excluded-study list was located; publication-bias tests were conditional on >=10 studies.	Evidence map
R13 Silva-Ribeiro et al. (22)	Low	7:No excluded-study list; 5/6:Only subsamples double-screened/checked	Pre-registered PRISMA/MARS review with eight databases, ROBINS-E/I and trim-and-fill. Screening/extraction were duplicated only for subsets and no excluded-study list was located.	Evidence map
R14 Tsoi et al. (23)	Critically Low	2:No protocol/registration located; 7:No excluded-study list located	Systematic literature review with JBI appraisal and Oxford levels. No protocol/registration or excluded-study list was located.	Evidence map
R15 Vandenbulcke et al. (24)	Low	7:No excluded-study list located	PROSPERO registration, PRISMA approach, comprehensive search and PEDro/JBI appraisal were reported. No excluded-study list was located; evidence remained narrative.	Core-eligible
R16 Wu et al. (25)	Critically Low	2:No protocol/registration; 7:No excluded-study list located	Comprehensive search and PEDro appraisal were reported, but no protocol/registration or excluded-study list was located; publication-bias assessment was not feasible/reported for small meta-analyses.	Core-eligible
R17 Yuen et al. (26)	Critically Low	2:No protocol/registration; 4:Limited search; 7:No excluded-study list; 9:No RoB appraisal	Fatigue management review with limited database search, no protocol, no duplicate process, no structured RoB appraisal, and no excluded-study list.	Evidence map
R18 Zouganeli et al. (27)	Critically Low	2:No protocol/registration located; 7:No excluded-study list located	PRISMA RCT-only review with comprehensive search and RoB 2. No protocol/registration or excluded-study list was located; no quantitative synthesis/certainty framework was reported.	Core-eligible

Overall confidence in the results of each review was rated with AMSTAR-2. A rating of High indicates no or one non-critical weakness; Moderate indicates more than one non-critical weakness but no critical weakness; Low indicates one critical weakness with or without non-critical weaknesses; Critically low indicates more than one critical weakness. Critical domains are items 2, 4, 7, 9, 11, 13 and 15. AMSTAR-2, A MeaSurement Tool to Assess systematic Reviews, version 2; RoB, risk of bias; SLE, systemic lupus erythematosus.

### Overlap among reviews

Overlap among the reviews was high to very high in every cluster. The overall CCA was 19.6% (very high), with cluster-specific values of 34.0% for structured exercise, 12.7% for physical activity and 14.3% for sedentary behavior. The largest pairwise overlaps were between Parodis et al. and Tsoi et al., between Blaess et al. and Tsoi et al., and between Blaess et al. and Parodis et al. (**Supplementary Data Sheet 1**: **Supplementary Table 5**; **Figure 3**). Because the reviews were therefore not statistically independent, their estimates are presented separately rather than pooled. Beyond pairwise overlap, the primary-study set of eight reviews was entirely contained within that of at least one other review. Wu et al. (25), which contributed three primary studies, was fully encapsulated by ten other reviews; O’Dwyer et al. (6) was fully contained within Ayán et al. (8); Parodis et al. (3) within Tsoi et al. (23); Santos et al. (5) within Vandenbulcke et al. (24); and Alexanderson and Boström (16), Del Pino-Sedeño et al. (18), Fangtham et al. (19) and Geertsema-Hoeve et al. (20) were each fully contained within one or more larger reviews. Only three reviews — Blaess et al. (17), Silva-Ribeiro et al. (22) and Tsoi et al. (23) — contributed any primary study that no other included review contained. The complete containment hierarchy is shown in **Supplementary Table 8**; **Supplementary Figure 1**.

### Effects on fatigue

For fatigue compared with usual or standard care, two of the three Core-eligible reviews reporting a pooled estimate found a statistically significant reduction favoring exercise: O’Dwyer et al. reported a mean difference (MD) of −0.61 (95% CI −1.19 to −0.02; 2 studies) (6) and Wu et al. an MD of −0.52 (95% CI −0.91 to −0.13; 3 studies) (25); Wu et al. additionally reported a pooled SF-36 vitality estimate from two studies favoring exercise (MD 14.98, 95% CI 7.45 to 22.52), shown separately because higher vitality scores indicate benefit. In contrast, the single high-confidence Cochrane review (Frade et al.) found no statistically significant difference for fatigue versus usual care (MD −0.59, 95% CI −1.40 to 0.22; I² = 0%) (7). Against an active comparator, the same Cochrane review reported a significant standardized reduction in fatigue (standardized mean difference [SMD] −0.51, 95% CI −0.88 to −0.14), whereas against placebo exercise the estimate was imprecise and non-significant (MD −5.00, 95% CI −13.29 to 3.29). Across these comparisons GRADE certainty was low (**Table 3**; **Figure 4A**). The O’Dwyer estimate pooled de Carvalho et al., 2005 and Tench et al., 2003 (115 analyzed participants); the Wu estimate added Ramsey-Goldman et al., 2000 to the same two trials (135 participants); and the Cochrane estimate pooled Tench et al., 2003 and Avaux et al., 2016 (104 participants). Thus, the three usual-care estimates drew on four unique primary trials. Counting each trial once and using the fullest relevant arm-level count reported across the source reviews, they represent 174 participants in the analyzed arms. The source reviews differed in their design labels for de Carvalho and Ramsey-Goldman and in the arm sizes extracted for Tench; **Supplementary Table 6** therefore preserves each source review’s classification and count rather than imposing a retrospective reclassification.

**Table 3:** Summary of meta-analysis findings from core-eligible reviews.

Review	Outcome (construct)	Comparator	Metric	No. of studies	Effect estimate [95% CI]	I²	P value	Certainty (GRADE)
Fatigue								
R06 Frade et al. (7)	Fatigue	Usual/standard care	MD	2	−0.59 [−1.40, 0.22]	0%	0.15	Low
R09 O'Dwyer et al. (6)	Fatigue	Usual/standard care	MD	2	−0.61 [−1.19, −0.02]	—	0.04	Low
R16 Wu et al. (25)	Fatigue	Usual/standard care	MD	3	−0.52 [−0.91, −0.13]	—	0.009	Low
R16 Wu et al. (25)	Fatigue/vitality a	Usual/standard care	MD	2	14.98 [7.45, 22.52]	—	—	Low
R06 Frade et al. (7)	Fatigue	Placebo exercise	MD	1	−5.00 [−13.29, 3.29]	—	0.24	Low
R06 Frade et al. (7)	Fatigue	Active comparator	SMD	2	−0.51 [−0.88, −0.14]	0%	0.007	Low
Disease activity								
R06 Frade et al. (7)	Disease activity	Usual/standard care	MD	2	−0.26 [−3.69, 3.17]	90%	0.88	Low
R09 O'Dwyer et al. (6)	Disease activity	Usual/standard care	MD	3	0.01 [−0.54, 0.56]	—	—	Low
R06 Frade et al. (7)	Disease activity	Active comparator	SMD	4	0.02 [−0.28, 0.32]	1%	0.87	Moderate
Withdrawals (any reason)								
R06 Frade et al. (7)	Withdrawals	Usual/standard care	RR	6	0.92 [0.53, 1.60]	0%	—	Very low
R06 Frade et al. (7)	Withdrawals	Placebo exercise	RR	1	2.73 [0.34, 22.16]	—	—	Very low
R06 Frade et al. (7)	Withdrawals	Active comparator	RR	7	0.89 [0.13, 5.94]	—	—	Very low

Each row is a single review’s pooled estimate for a clinical construct against the stated comparator. Estimates from different reviews are presented separately and are not combined, because the reviews draw on substantially overlapping primary studies (see **Supplementary Data Sheet 1**: **Supplementary Table 5**; **Figure 3**) and report heterogeneous metrics and comparators; pooling across them would count the same trials more than once. Only Core-eligible reviews are shown. Metrics are not mixed within a comparison. A negative mean difference or standardized mean difference favors exercise for fatigue and disease activity (lower scores are better). For withdrawals, a risk ratio below 1 favors exercise. CI, confidence interval; GRADE, Grading of Recommendations Assessment, Development and Evaluation; I², percentage of variability across studies due to heterogeneity; MD, mean difference; RR, risk ratio; SMD, standardized mean difference; —, not reported or not applicable.

### Effects on disease activity

Disease activity was not significantly changed by exercise in any Core-eligible comparison. Versus usual care, O’Dwyer et al. reported an MD of 0.01 (95% CI −0.54 to 0.56) (6) and Frade et al. an MD of −0.26 (95% CI −3.69 to 3.17) with substantial heterogeneity (I² = 90%) (7). Versus an active comparator, Frade et al. reported an SMD of 0.02 (95% CI −0.28 to 0.32; I² = 1%). Certainty was low for the comparison with usual care and moderate for the comparison with an active comparator, the latter being the only outcome rated above low certainty in the review (**Table 3**; **Figure 4B**). The usual-care estimates drew on five primary trials in total: 127 participants across three trials in O’Dwyer et al. and 100 participants across two trials in the Cochrane review. The Cochrane active-comparator estimate included four trials and 184 participants (**Supplementary Table 6**).

### Tolerability and safety

The Cochrane withdrawal comparisons were statistically inconclusive: the risk ratio (RR) was 0.92 (95% CI 0.53 to 1.60) versus usual care, 2.73 (95% CI 0.34 to 22.16) versus placebo exercise and 0.89 (95% CI 0.13 to 5.94) versus an active comparator, all with very low certainty owing to very few events and wide confidence intervals (7). These estimates do not establish equivalence or exclude clinically important benefit or harm. Serious adverse events and disease flares were reported only narratively across the Core-eligible reviews and did not reveal a consistent safety signal; these outcomes were not meta-analyzed and their certainty was not formally graded. Withdrawal estimates are summarized in **Table 3**. Because flares were defined inconsistently and were not prospectively monitored in most contributing trials, the narrative absence of a signal should be interpreted as absence of evidence of harm, not evidence of absence of harm.

### Sensitivity synthesis by methodological confidence

Restricting the Core-eligible evidence to reviews rated high or low overall confidence on AMSTAR-2 removed Blaess et al. (17), Wu et al. (25) and Zouganeli et al. (27) from the primary synthesis. For fatigue versus usual or standard care this left two pooled estimates, from O’Dwyer et al. (6), which remained statistically significant, and from Frade et al. (7), which did not; one of the two significant fatigue estimates therefore no longer contributed. Restricting further to the single high-confidence review left no statistically significant difference in fatigue versus usual care, a significant standardized reduction versus an active comparator, no statistically significant disease-activity difference, and withdrawal estimates of very low certainty. The direction of the disease-activity and withdrawal findings was unchanged in both restricted sets. The confidence-restricted synthesis therefore supports a more conservative reading of the fatigue evidence than the unrestricted synthesis, and this is reflected in the Discussion and Conclusions (**Supplementary Table 7**).

### Other outcomes and observational associations

Other outcomes — aerobic capacity, health-related quality of life, depression and pain — were reported inconsistently and largely narratively across reviews, with several reviews suggesting possible improvements in aerobic capacity and quality of life but without pooled SLE-specific estimates that would support a graded conclusion. Inflammatory and immune markers were similarly addressed only narratively. Among evidence-map reviews, a meta-analysis of observational data [Silva-Ribeiro et al. (22)] found that higher physical activity was associated with lower disease activity (pooled estimate −0.299, 95% CI −0.473 to −0.124; 6 studies) and with lower fatigue and pain (−0.227, 95% CI −0.340 to −0.114; 9 studies); these associations were provisionally classified as Class IV (the weakest category) because the source review did not report all inputs required to verify Classes I–III; the contributing studies were observational and predominantly at high risk of bias. A construct-level summary of the direction of effect and certainty is provided in **Table 4** and **Figure 5**, with review-level details in **Supplementary Data Sheet 1**: **Supplementary Table 4**.

**Table 4:** Certainty of the evidence by outcome and comparator.

Outcome (construct)	Comparator	Framework	Contributing reviews	Certainty	Comment
Intervention comparisons (Core-eligible)					
Fatigue	Usual/standard care	GRADE	3 (Frade, O’Dwyer, Wu)	Low	O’Dwyer and Wu reported a significant reduction; the Cochrane review (Frade) found no significant difference. Downgraded for risk of bias and imprecision.
Fatigue	Placebo exercise	GRADE	1 (Frade)	Low	Statistically inconclusive; confidence interval includes clinically important benefit and harm.
Fatigue	Active comparator	GRADE	1 (Frade)	Low	Favors exercise (SMD −0.51); few trials.
Disease activity	Usual/standard care	GRADE	2 (Frade, O’Dwyer)	Low	No statistically significant difference; the Cochrane estimate was imprecise and substantially heterogeneous (I² = 90%).
Disease activity	Active comparator	GRADE	1 (Frade)	Moderate	Probably little to no difference; estimate is precise around the null.
Withdrawals (any reason)	Usual/standard care	GRADE	1 (Frade)	Very low	Statistically inconclusive; very serious imprecision (few events).
Withdrawals (any reason)	Placebo exercise	GRADE	1 (Frade)	Very low	Statistically inconclusive; very few events and a very wide confidence interval.
Withdrawals (any reason)	Active comparator	GRADE	1 (Frade)	Very low	Statistically inconclusive; very few events and a very wide confidence interval.
Observational exposure associations (evidence map only)					
Disease/illness activity	Reference/lower physical activity	Ioannidis classification	1 (Silva-Ribeiro)	Class IV*	Observational exposure; weak inverse association; provisionally Class IV because criteria for Classes I–III could not be verified from the source report; most contributing studies were at high risk of bias.
Fatigue and pain (composite)	Reference/lower physical activity	Ioannidis classification	1 (Silva-Ribeiro)	Class IV*	Observational composite outcome; weak inverse association; provisionally Class IV because criteria for Classes I–III could not be verified from the source report.

Certainty was assessed with GRADE for intervention comparisons and with a provisional Ioannidis classification for observational exposure associations; where a Core-eligible review reported its own GRADE rating it was retained. For overview-applied GRADE ratings, two reviewers independently considered risk of bias, inconsistency, indirectness, imprecision and publication bias. Ratings are summarized at the level of the clinical construct and comparator rather than for individual scales. Where contributing reviews differed, the most cautious rating is shown and the difference is described in the comment.

GRADE certainty reflects how much confidence can be placed in the estimate: High, Moderate, Low or Very low. Provisional Ioannidis Class IV denotes the weakest category and indicates that the source report did not provide sufficient information to verify Classes I–III. CI, confidence interval; GRADE, Grading of Recommendations Assessment, Development and Evaluation; I², heterogeneity statistic; SMD, standardized mean difference; SLE, systemic lupus erythematosus.

## Discussion

In this umbrella review of 18 systematic reviews, exercise and physical activity were associated with small reductions in fatigue compared with usual care in two of three Core-eligible reviews (6, 25), but the single high-confidence Cochrane review did not find a statistically significant fatigue benefit (7), and overall GRADE certainty was low. Disease-activity estimates were not statistically significant in the available pooled comparisons (6, 7). Withdrawal comparisons were statistically inconclusive and of very low certainty, while serious adverse events and disease flares were available only as narrative, ungraded evidence (7, 8, 17, 24, 27). Taken together, the evidence is compatible with a modest favorable effect on fatigue, but it does not establish tolerability, an immune-inflammatory mechanism or a disease-modifying effect.

Several factors explain the discordance among reviews. The reviews used different fatigue scales, comparators and analytical methods, included different (though heavily overlapping) sets of primary trials, and varied substantially in methodological quality (6–8, 24, 25, 27). The statistically significant fatigue estimates came largely from low-confidence reviews (6, 25), whereas the most rigorous review — a Cochrane review with a registered protocol, duplicate processes, a list of excluded studies and GRADE summary-of-findings tables — reached more conservative conclusions (7). The very high overlap among reviews (overall CCA 19.6%), quantified using the corrected covered area method (15), further means that apparently consistent findings often reflect the same underlying trials counted more than once, which is precisely why we refrained from pooling across reviews.

Two features of this literature are easily missed when the reviews are read individually. First, several reviews report estimates that were not independently derived but restated from an earlier review. The fatigue and disease-activity estimates in Parodis et al. (3), the systematic literature review informing the 2023 EULAR recommendations for the non-pharmacological management of SLE, are the pooled estimates of O’Dwyer et al. (6) and Wu et al. (25) reproduced unchanged. Gwinnutt et al. (4) restate the same point estimates, with a minor rounding difference in the lower confidence limit for the Wu fatigue estimate (−0.92 rather than −0.91). Matching point estimates with identical or near-identical confidence intervals in reviews whose included-study lists differ are therefore not independent corroboration; they are one analysis appearing more than once, and readers who count reviews rather than trials will overestimate the weight of the evidence. Second, the primary evidence base is very small. Every pooled fatigue estimate for exercise versus usual or standard care in this field rests on four primary trials representing 174 participants in the relevant analyzed arms when each trial is counted once, with no single pooled estimate including more than 135 participants and one contributing trial enrolling only ten. The corresponding disease-activity estimates rest on five trials and no more than 184 participants in any single comparison.

The contributing trials are themselves heterogeneous in ways that review-level reporting tends to conceal. They used different comparators (no intervention, relaxation therapy, education and non-exercise control), different exercise modes, doses and settings (supervised versus home-based programs of 8 to 12 weeks), and different fatigue instruments (Fatigue Severity Scale, Chalder Fatigue Scale, fatigue visual analogue scale and the SF-36 vitality domain), so estimates presented as addressing ‘fatigue versus usual care’ in fact address several different questions. The source reviews also disagreed about the trials themselves: de Carvalho et al., 2005 and Ramsey-Goldman et al., 2000 were treated as randomized by some reviews and as quasi-randomized or quasi-experimental by others, and the arm sizes extracted for Tench et al., 2003 differed between reviews. Review-level discordance therefore partly reflects unresolved heterogeneity and inconsistent extraction at trial level, not only differences in review methods (**Supplementary Table 6**).

These findings are consistent with guideline recommendations and recommendation-informing reviews that endorse exercise and physical activity as non-pharmacological adjuncts in the management of SLE and other inflammatory rheumatic diseases (3–5), and with broader evidence in rheumatic and musculoskeletal diseases suggesting a moderate effect of aerobic exercise on fatigue (4, 5). At the same time, the umbrella-level appraisal makes clear that the SLE-specific evidence is limited, derived from few and often small primary trials, and dominated by reviews of low or critically low methodological quality (6–8, 24, 25, 27). The observational associations between higher physical activity and lower disease activity and fatigue are encouraging (22), but, being provisionally classified as Class IV, cannot establish causal benefit.

### Immune-inflammatory interpretation

An immunological interpretation of the fatigue findings is most defensible as a mechanistic hypothesis rather than as a proven mediation pathway. Exercise could plausibly influence SLE fatigue through interconnected peripheral and central pathways: repeated muscle contraction induces myokine responses and, over time, may reduce chronic low-grade inflammation; improved fitness and body composition may blunt adiposity-related IL-6/CRP signaling; and reduced inflammatory signaling may attenuate sickness-behavior pathways relevant to fatigue and sleep disturbance (33–36). SLE-specific data are consistent with, but do not confirm, this model. In women with SLE, higher cardiorespiratory fitness attenuated the association of adiposity with IL-6 and CRP, and a randomized trial found that moderate aerobic training improved insulin sensitivity with increased skeletal-muscle AMPK expression, but neither study established that immune modulation mediated fatigue improvement or disease-activity change (37, 38).

The main immunology gap is therefore not the absence of biological plausibility, but the absence of integrated mechanistic testing in the SLE exercise literature represented by the included reviews. Those reviews did not provide pooled data for type I interferon signatures, complement activation, anti-dsDNA antibodies, Th17/Treg balance, neutrophil extracellular traps, B-cell activation or cytokine panels, despite the relevance of these pathways to lupus pathogenesis (39). Absence at review level is not the same as absence at trial level. Several primary studies included in the reviews measured high-sensitivity C-reactive protein, IL-6, TNF-α and other cytokines, and one randomized trial reported skeletal-muscle AMPK expression together with insulin sensitivity (7, 17, 38). Within the primary studies represented by the included reviews, however, we found no exercise trial that prespecified a type I interferon gene signature, complement activation, anti-dsDNA titres, Th17/Treg balance, neutrophil extracellular trap markers or B-cell activation as an endpoint.

An SLE-specific caveat applies to the myokine and anti-inflammatory framework invoked above, which derives largely from healthy or metabolically diseased populations rather than from a chronically interferon-activated immune system (33–36). Experimental work shows that interferon-α can suppress hepatocyte production of C-reactive protein (40), and in patients with SLE CRP was positively associated with detectable IL-6 but negatively associated with a type I interferon gene signature (41). Two implications follow. First, CRP, and by extension the IL-6/CRP axis commonly used to index the anti-inflammatory effect of exercise, may be an unreliable readout of inflammatory tone in interferon-high SLE. Second, a chronically interferon-driven environment may blunt training adaptation, so an inflammation-mediated fatigue benefit could be smaller in interferon-high than in interferon-low patients. This is a testable hypothesis rather than an established mechanism, and it supports stratifying future exercise trials by interferon status.

Heterogeneity of SLE itself is a further, underrecognized source of variability. Incidence, prevalence, ancestral composition, organ involvement, treatment access and survival differ substantially between world regions and have changed over the period spanned by the trials synthesized here (42); within any cohort, patients also differ in disease duration, damage accrual, glucocorticoid and immunosuppressive exposure, serological activity and comorbidity. Trials conducted in different decades and settings therefore recruit clinically and immunologically different populations under the same diagnostic label. This is a plausible contributor to inconsistent primary-study findings and discordant review estimates, and it reinforces the need to report and, where possible, stratify by disease-related characteristics rather than presenting an average effect for ‘SLE’ as a homogeneous condition.

Future SLE trials should embed mechanistic endpoints alongside patient-reported fatigue, SLEDAI/BILAG-based disease activity, medication exposure and flare surveillance. A minimal immune-inflammatory panel should include high-sensitivity CRP, IL-6, TNF-α, IL-10, complement C3/C4, anti-dsDNA antibodies and an interferon-stimulated gene score, with blood sampling standardized relative to the last exercise session and to glucocorticoid or immunosuppressive dosing. Larger trials could add B-cell and T-cell immunophenotyping, Treg/Th17 balance, neutrophil extracellular trap markers and adipokines. Recent randomized trials indicate that some of these gaps are beginning to be addressed. The Living Well with Lupus lifestyle trial evaluated a structured lifestyle intervention with cardiovascular risk-related outcomes (43), while a more recent trial examined combined progressive resistance and aerobic exercise in patients with osteoporotic SLE (44). The 53-participant LUPEX trial further extended this evidence by evaluating high-intensity interval training together with an IFN-I-regulated gene-expression score; aerobic capacity improved, whereas fatigue did not, and the observed IFN-I-related differences in training response were exploratory (45). Collectively, these studies suggest increasing attention to structured exercise prescription and mechanistically informative outcomes, but they also reinforce the need for adequately powered trials integrating standardized fatigue and disease-activity measures, prospective flare surveillance, medication exposure and prespecified immune-inflammatory endpoints.

This review has several strengths. It used a comprehensive, multi-database search reported according to PRISMA-S (13) and aligned with PRISMA 2020 and PRIOR reporting guidance (11, 12); study selection and data extraction were conducted independently by two reviewers; every included review was systematically appraised with AMSTAR-2 (14); overlap was quantified with the corrected covered area (15), and review estimates were not pooled across overlapping evidence bases; certainty was graded using GRADE (28) and the Ioannidis framework for umbrella-level evidence appraisal (9); and intervention evidence was separated from observational associations and Core-eligible reviews from evidence-map reviews. Because Core eligibility was defined by relevance rather than methodological quality, we also report a confidence-restricted sensitivity synthesis and a study-level account of the trials and participants underlying every pooled estimate.

Important limitations should be acknowledged. First, this overview necessarily depends on how the source reviews classified and extracted their primary trials; discrepancies in design labels and arm sizes could not always be reconciled without re-analysis of the original trial datasets. Second, the very high overlap among reviews means that the reviews are not independent, which constrained synthesis and precluded meta-analysis at the review level. Third, most included reviews were of low or critically low confidence under AMSTAR-2, chiefly because a registered protocol and a list of excluded studies were not located. Fourth, the underlying primary evidence comprises relatively few and often small trials with heterogeneous comparators, outcomes and fatigue instruments. Fifth, although this overview was registered, a separate full protocol was not published. Sixth, inflammatory or immune markers, disease flares and sedentary behavior were under-represented in quantitative evidence and could be summarized only narratively. Finally, Core eligibility was defined by relevance rather than methodological quality; conclusions from that tier are therefore sensitive to critically low-confidence reviews and should be read alongside the confidence-restricted synthesis.

For clinical practice, the findings support discussing individualized exercise and physical activity as adjuncts, delivered alongside rather than instead of standard pharmacological care, because they may help some adults with SLE to reduce fatigue (3–5, 7). The reviews identified no consistent safety signal, but the very-low-certainty withdrawal data and sparse, ungraded flare evidence do not establish safety or tolerability. Exercise should therefore be introduced and progressed with attention to current disease activity, organ involvement, comorbidity, medication exposure and prospective monitoring of flares and adverse events. For research, adequately powered randomized trials are needed with standardized fatigue and disease-activity outcomes, longer follow-up, prespecified flare surveillance and immune-inflammatory endpoints, and complete reporting of exercise dose, intensity, adherence, medication changes, baseline disease activity, body composition, sleep and mood. Future systematic reviews should be prospectively registered, report excluded-study lists, avoid unnecessary duplication and distinguish mechanistic outcomes from clinical patient-reported outcomes.

## Conclusions

Exercise and physical activity may produce small reductions in fatigue in adults with SLE, but the review-level evidence is low-certainty and substantially overlapping. The available reviews identified no consistent safety signal; nevertheless, withdrawal estimates were of very low certainty and evidence on disease flares was sparse, narrative and ungraded, so safety and tolerability remain uncertain. Disease-activity estimates were not statistically significant, and the evidence does not establish an immune-inflammatory mechanism. The usual-care fatigue signal ultimately derives from four small primary trials representing 174 participants in the relevant analyzed arms when each trial is counted once, several of which are reused across reviews and guideline-informing documents. New, adequately powered primary trials with standardized clinical outcomes, prospective flare monitoring and prespecified immunological endpoints are therefore a higher priority than further reviews of the same limited evidence base.
