# Supplementary material for: Exercise and physical activity for fatigue, disease activity and immune-inflammatory outcomes in adults with systemic lupus erythematosus: an umbrella review
Source: Front Immunol. 2026 Sep 18;17:1925582. doi: 10.3389/fimmu.2026.1925582 (PMC13631844; doi:10.3389/fimmu.2026.1925582)
Supplement: Supplementary file 1 [file Table1.docx]

# Supplementary

Contents: Table S1, complete database search strategies and dated record counts; Table S2, outcome-level evidence from all included reviews; Table S3, AMSTAR-2 methodological quality assessments; Table S4, certainty of the evidence; Table S5, primary-study overlap (corrected covered area); Table S6, primary studies and participants contributing to each pooled estimate; Table S7, sensitivity synthesis restricted by AMSTAR-2 confidence; Table S8, complete containment of primary-study sets between reviews; Supplementary Figure S1, containment hierarchy (supplied as a separate file). Main-manuscript figures are numbered Figures 1–5.

## Table S1. Complete search strategies for all electronic databases.

| **Database** | **Executable search string** |
| --- | --- |
| PubMed (MEDLINE) Records retrieved: n = 97 | #1 Population  "Lupus Erythematosus, Systemic"[Mesh] OR "systemic lupus erythematosus"[tiab] OR "lupus erythematosus"[tiab] OR SLE[tiab] OR lupus[tiab]  #2 Intervention/exposure  "Exercise"[Mesh] OR "Exercise Therapy"[Mesh] OR "Physical Fitness"[Mesh] OR "Physical Conditioning, Human"[Mesh] OR "Resistance Training"[Mesh] OR "Sedentary Behavior"[Mesh] OR exercise*[tiab] OR "physical activity"[tiab] OR "physical training"[tiab] OR "physical fitness"[tiab] OR aerobic*[tiab] OR "resistance training"[tiab] OR "strength training"[tiab] OR "muscle strengthening"[tiab] OR walking[tiab] OR "aerobic capacity"[tiab] OR "sedentary behavior"[tiab] OR "sitting time"[tiab] OR "physical inactivity"[tiab]  #3 Study-design filter  "systematic review"[pt] OR "meta-analysis"[pt] OR "systematic review"[tiab] OR "meta-analysis"[tiab] OR "meta analysis"[tiab] OR metaanalys*[tiab] OR (systematic[tiab] AND review[tiab]) OR "umbrella review"[tiab] OR "overview of reviews"[tiab]  #4 Combined  #1 AND #2 AND #3 |
| Embase (Elsevier Embase.com) Records retrieved: n = 247 | #1  'systemic lupus erythematosus'/exp OR 'systemic lupus erythematosus':ti,ab OR 'lupus erythematosus':ti,ab OR sle:ti,ab OR lupus:ti,ab  #2  'exercise'/exp OR 'kinesiotherapy'/exp OR 'fitness'/exp OR 'sedentary lifestyle'/exp OR 'resistance training'/exp OR exercise*:ti,ab OR 'physical activity':ti,ab OR 'physical training':ti,ab OR 'physical fitness':ti,ab OR aerobic*:ti,ab OR 'resistance training':ti,ab OR 'strength training':ti,ab OR 'muscle strengthening':ti,ab OR walking:ti,ab OR 'aerobic capacity':ti,ab OR 'sedentary behavior':ti,ab OR 'sitting time':ti,ab OR 'physical inactivity':ti,ab  #3  'systematic review'/exp OR 'meta analysis'/exp OR 'systematic review':ti,ab OR 'meta-analysis':ti,ab OR 'meta analysis':ti,ab OR metaanalys*:ti,ab OR (systematic:ti,ab AND review:ti,ab) OR 'umbrella review':ti,ab OR 'overview of reviews':ti,ab  #4  #1 AND #2 AND #3 |
| Cochrane Library (CDSR) Records retrieved: n = 2 | #1 [mh "Lupus Erythematosus, Systemic"] OR (systemic NEXT lupus):ti,ab,kw OR SLE:ti,ab,kw OR lupus:ti,ab,kw #2 [mh Exercise] OR [mh "Exercise Therapy"] OR [mh "Physical Fitness"] OR [mh "Sedentary Behavior"] OR [mh "Resistance Training"] OR exercise*:ti,ab,kw OR (physical NEXT activity):ti,ab,kw OR aerobic*:ti,ab,kw OR (resistance NEXT training):ti,ab,kw OR (strength NEXT training):ti,ab,kw OR walking:ti,ab,kw OR (sedentary NEXT behavi*):ti,ab,kw OR (sitting NEXT time):ti,ab,kw #3 Combined #1 AND #2 Note The search was run in the Cochrane Database of Systematic Reviews. The reported n=2 therefore represents review records only; CENTRAL trial records were not included in the screening dataset. |
| Web of Science Core Collection Records retrieved: n = 161 | #1  TS=("systemic lupus erythematosus" OR "lupus erythematosus" OR SLE OR lupus)  #2  TS=(exercise* OR "physical activity" OR "physical training" OR "physical fitness" OR aerobic* OR "resistance training" OR "strength training" OR "muscle strengthening" OR walking OR "aerobic capacity" OR "sedentary behavior" OR "sitting time" OR "physical inactivity")  #3  TS=("systematic review" OR "meta-analysis" OR "meta analysis" OR metaanalys* OR "umbrella review" OR "overview of reviews")  #4 Combined  #1 AND #2 AND #3 |
| Scopus Records retrieved: n = 179 | #1  TITLE-ABS-KEY("systemic lupus erythematosus" OR "lupus erythematosus" OR SLE OR lupus)  #2  TITLE-ABS-KEY(exercise* OR "physical activity" OR "physical training" OR "physical fitness" OR aerobic* OR "resistance training" OR "strength training" OR "muscle strengthening" OR walking OR "aerobic capacity" OR "sedentary behavior" OR "sitting time" OR "physical inactivity")  #3  TITLE-ABS-KEY("systematic review" OR "meta-analysis" OR "meta analysis" OR metaanalys* OR "umbrella review" OR "overview of reviews")  #4 Combined  #1 AND #2 AND #3 |
| SPORTDiscus (EBSCOhost) Records retrieved: n = 2 | #1  (MH "Systemic Lupus Erythematosus") OR TI("systemic lupus erythematosus" OR "lupus erythematosus" OR SLE OR lupus) OR AB("systemic lupus erythematosus" OR "lupus erythematosus" OR SLE OR lupus)  #2  (MH "Exercise+") OR (MH "Physical Activity") OR (MH "Sedentary Behavior") OR TI(exercise* OR "physical activity" OR aerobic* OR "resistance training" OR "strength training" OR walking OR "sedentary behavio#r" OR "sitting time") OR AB(exercise* OR "physical activity" OR aerobic* OR "resistance training" OR "strength training" OR walking OR "sedentary behavio#r" OR "sitting time")  #3  TI("systematic review" OR "meta-analysis" OR "meta analysis" OR "umbrella review") OR AB("systematic review" OR "meta-analysis" OR "meta analysis")  #4 Combined  #1 AND #2 AND #3 |

All databases were searched on 1 March 2026. Records retrieved in each dated export are given in the first column and total 688. For the Cochrane Library, the reported count comprises Cochrane Database of Systematic Reviews records only (n = 2); CENTRAL trial records were not included in the count or screening dataset because primary trial reports were ineligible under the study-design criterion. Dated exports, de-duplication records and the two-reviewer screening files are available from the corresponding authors on reasonable request.

## Table S2. Outcome-level evidence from all included reviews.

| **Outcome (construct)** | **Comparator** | **Reported finding** |
| --- | --- | --- |
| R01 — Alexanderson et al. (2020) · Evidence-map only | | |
| Fatigue | Usual / standard care | Narrative summary; no pooled estimate reported. |
| Disease activity | Usual / standard care | Narrative summary; no pooled estimate reported. |
| Inflammatory/immune markers | Usual / standard care | Narrative summary; no pooled estimate reported. |
| R02 — Ayán et al. (2018) · Core-eligible | | |
| Fatigue | Usual / standard care | Narrative summary; no pooled estimate reported. |
| Disease activity | Usual / standard care | Narrative summary; no pooled estimate reported. |
| Safety/adverse events | Usual / standard care | Narrative summary; no pooled estimate reported. |
| R03 — Blaess et al. (2023) · Core-eligible | | |
| Fatigue | Reference / lower exposure | Narrative summary; no pooled estimate reported. |
| Disease activity | Reference / lower exposure | Narrative summary; no pooled estimate reported. |
| Inflammatory/immune markers | Reference / lower exposure | Narrative summary; no pooled estimate reported. |
| Safety/adverse events | Reference / lower exposure | Narrative summary; no pooled estimate reported. |
| R04 — Del Pino-Sedeño et al. (2016) · Evidence-map only | | |
| Fatigue | Usual / standard care | Narrative summary; no pooled estimate reported. |
| Fatigue | Usual / standard care | Narrative: VAS fatigue significant reduction; Cohen d 0.83-1.68; effect size 0.37-0.63 |
| Fatigue | Active comparator | Narrative: CFS effect size 0.89; VAS effect size 0.83 |
| Fatigue | Usual / standard care | Narrative: Cohen d 0.63; effect size 0.30 |
| Fatigue | Usual / standard care | Narrative: 18.4% reduction from baseline; Cohen d 1.05; effect size 0.47 |
| R05 — Fangtham et al. (2019) · Evidence-map only | | |
| Fatigue | Usual / standard care | Narrative summary; no pooled estimate reported. |
| Disease activity | Usual / standard care | Narrative summary; no pooled estimate reported. |
| Safety/adverse events | Usual / standard care | Narrative summary; no pooled estimate reported. |
| R06 — Frade et al. (2023) · Core-eligible | | |
| Fatigue | Placebo exercise | MD −5.00 [−13.29, 3.29] (k = 1) |
| Withdrawals | Placebo exercise | RR 2.73 [0.34, 22.16] (k = 1) |
| Fatigue | Usual / standard care | MD −0.59 [−1.40, 0.22] (I² = 0%; k = 2) |
| Disease activity | Usual / standard care | MD −0.26 [−3.69, 3.17] (I² = 90%; k = 2) |
| Withdrawals | Usual / standard care | RR 0.92 [0.53, 1.60] (I² = 0%; k = 6) |
| Fatigue | Active comparator | SMD −0.51 [−0.88, −0.14] (I² = 0%; k = 2) |
| Disease activity | Active comparator | SMD 0.02 [−0.28, 0.32] (I² = 1%; k = 4) |
| Withdrawals | Active comparator | RR 0.89 [0.13, 5.94] (k = 7) |
| R07 — Geertsema-Hoeve et al. (2024) · Evidence-map only | | |
| Disease activity | Usual / standard care | Narrative summary; no pooled estimate reported. |
| Fatigue | Usual / standard care | Narrative: Exercise 2.7 (1.3) vs control 3.3 (1.5) as reported |
| Fatigue | Active comparator | Narrative: 6.63 (7.15) vs -0.71 (9.05) as reported Higher-is-better (vitality-type) scale; direction opposite to the fatigue scales. |
| R08 — Gwinnutt et al. (2022) · Evidence-map only | | |
| Fatigue | Usual / standard care | MD −0.61 [−1.19, −0.02] Re-reports the O’Dwyer pooled estimate; shares primary studies. |
| Fatigue | Usual / standard care | MD −0.52 [−0.92, −0.13]. Re-reports the Wu pooled estimate with a minor rounding difference in the lower confidence limit (Wu: −0.91); shares primary studies. |
| Disease activity | Usual / standard care | Narrative summary; no pooled estimate reported. |
| Fatigue | Alternative exercise | Narrative summary; no pooled estimate reported. |
| R09 — O'Dwyer et al. (2017) · Core-eligible | | |
| Disease activity | Usual / standard care | MD 0.01 [−0.54, 0.56] (k = 3) |
| Fatigue | Usual / standard care | MD −0.61 [−1.19, −0.02] (k = 2) |
| Fatigue | Active comparator | MD −6.0 [−10.3, −1.7] (k = 1) Single primary-study result reported within the review, not a review-level pooled estimate. |
| Safety/adverse events | Usual / standard care | Narrative summary; no pooled estimate reported. |
| R10 — Parodis et al. (2023) · Evidence-map only | | |
| Fatigue | Usual / standard care | MD −0.61 [−1.19, −0.02] Re-reports the O’Dwyer pooled estimate; shares primary studies. |
| Fatigue | Usual / standard care | MD −0.52 [−0.91, −0.13] Re-reports the Wu pooled estimate; shares primary studies. |
| Disease activity | Usual / standard care | MD 0.01 [−0.54, 0.56] Re-reports the O’Dwyer pooled estimate; shares primary studies. |
| R11 — Rodríguez Huerta et al. (2016) · Evidence-map only | | |
| Fatigue | Usual / standard care | Narrative summary; no pooled estimate reported. |
| Fatigue | Active comparator | Narrative: CFS significant reduction; self-perceived improvement exercise 49% vs relaxation 28% vs control 16% |
| Fatigue | Usual / standard care | Narrative: 3.57 ± 1.47 to 2.68 ± 1.33 |
| Fatigue | Usual / standard care | Narrative: VAS improved; POMS not significant |
| Disease activity | Usual / standard care | Narrative summary; no pooled estimate reported. |
| R12 — Santos et al. (2023) · Evidence-map only | | |
| Fatigue | Usual / standard care | SMD −0.54 [−1.07, −0.01] (I² = <75%) |
| R13 — Silva-Ribeiro et al. (2025) · Evidence-map only | | |
| Disease activity | Reference / lower exposure | Association coefficient −0.299 [−0.473, −0.124] (k = 6) |
| Fatigue and pain | Reference / lower exposure | Association coefficient −0.227 [−0.340, −0.114] (k = 9) |
| Disease activity | Reference / lower exposure | Narrative summary; no pooled estimate reported. |
| R14 — Tsoi et al. (2024) · Evidence-map only | | |
| Fatigue | Usual / standard care | Narrative summary; no pooled estimate reported. |
| Disease activity | Usual / standard care | Narrative summary; no pooled estimate reported. |
| R15 — Vandenbulcke et al. (2023) · Core-eligible | | |
| Fatigue | Usual / standard care | Narrative summary; no pooled estimate reported. |
| Disease activity | Usual / standard care | Narrative summary; no pooled estimate reported. |
| R16 — Wu et al. (2017) · Core-eligible | | |
| Fatigue | Usual / standard care | MD −0.52 [−0.91, −0.13] (k = 3) |
| Fatigue | Usual / standard care | MD 14.98 [7.45, 22.52] (k = 2). SF-36 vitality (higher scores better); not combined with lower-is-better fatigue scales. |
| R17 — Yuen et al. (2014) · Evidence-map only | | |
| Fatigue | Usual / standard care | Narrative summary; no pooled estimate reported. |
| Disease activity | Usual / standard care | Narrative summary; no pooled estimate reported. |
| R18 — Zouganeli et al. (2025) · Core-eligible | | |
| Fatigue | Usual / standard care | Narrative summary; no pooled estimate reported. |
| Disease activity | Usual / standard care | Narrative summary; no pooled estimate reported. |
| Safety/adverse events | Usual / standard care | Narrative summary; no pooled estimate reported. |

Findings are shown for every included review and are grouped by review. Quantitative cells give the metric, point estimate and 95% confidence interval as reported; heterogeneity (I²) and the number of contributing primary studies (k) are shown where available. Narrative cells summarize the direction of evidence where the review did not report a pooled estimate.

Interpretation of direction: for fatigue and disease activity a negative mean difference or standardized mean difference favors exercise; for withdrawals a risk ratio below 1 favors exercise; vitality-type scales are scored in the opposite direction, as noted.

Abbreviations: CI, confidence interval; I², heterogeneity statistic; k, number of primary studies; MD, mean difference; RR, risk ratio; SF-36, 36-item Short-Form Health Survey; SMD, standardized mean difference.

## Table S3. AMSTAR-2 item-level methodological quality assessments.

| **Review** | **1** | **2** | **3** | **4** | **5** | **6** | **7** | **8** | **9** | **10** | **11** | **12** | **13** | **14** | **15** | **16** | **Overall** |
| --- | --- | --- | --- | --- | --- | --- | --- | --- | --- | --- | --- | --- | --- | --- | --- | --- | --- |
| R01 Alexanderson et al. | Y | N | PY | PY | N | PY | N | Y | Y | N | NA | NA | PY | PY | NA | Y | C. low |
| R02 Ayán et al. | Y | Y | Y | PY | PY | PY | N | Y | Y | N | NA | NA | PY | PY | NA | N | Low |
| R03 Blaess et al. | Y | N | PY | N | Y | PY | N | Y | N | N | NA | NA | N | PY | NA | Y | C. low |
| R04 Del Pino-Sedeño et al. | Y | N | Y | Y | Y | PY | N | Y | Y | N | NA | NA | Y | Y | NA | Y | C. low |
| R05 Fangtham et al. | Y | N | PY | Y | PY | N | N | Y | N | N | NA | NA | N | Y | NA | Y | C. low |
| R06 Frade et al. | Y | Y | Y | Y | Y | Y | Y | Y | Y | PY | Y | Y | Y | Y | PY | Y | High |
| R07 Geertsema-Hoeve et al. | Y | N | Y | PY | Y | PY | N | Y | Y | N | NA | NA | PY | Y | NA | Y | C. low |
| R08 Gwinnutt et al. | Y | N | Y | Y | PY | PY | N | Y | Y | PY | PY | PY | Y | Y | PY | Y | C. low |
| R09 O'Dwyer et al. | Y | Y | Y | Y | Y | PY | N | Y | Y | N | Y | PY | Y | PY | PY | Y | Low |
| R10 Parodis et al. | Y | N | Y | Y | Y | Y | N | Y | PY | N | NA | NA | PY | Y | NA | Y | C. low |
| R11 Rodríguez Huerta et al. | Y | N | Y | Y | Y | PY | N | Y | Y | N | NA | NA | Y | Y | NA | Y | C. low |
| R12 Santos et al. | Y | Y | Y | Y | Y | Y | N | Y | Y | N | Y | Y | Y | Y | PY | Y | Low |
| R13 Silva-Ribeiro et al. | Y | Y | Y | Y | PY | PY | N | Y | Y | N | Y | Y | Y | Y | Y | Y | Low |
| R14 Tsoi et al. | Y | N | Y | Y | Y | Y | N | Y | Y | N | NA | NA | Y | Y | NA | Y | C. low |
| R15 Vandenbulcke et al. | Y | Y | Y | Y | Y | PY | N | Y | Y | N | NA | NA | Y | Y | NA | Y | Low |
| R16 Wu et al. | Y | N | Y | Y | PY | PY | N | Y | Y | N | Y | PY | Y | PY | N | Y | C. low |
| R17 Yuen et al. | PY | N | PY | N | N | N | N | PY | N | N | NA | NA | N | PY | NA | Y | C. low |
| R18 Zouganeli et al. | Y | N | Y | Y | Y | PY | N | Y | Y | N | NA | NA | Y | Y | NA | Y | C. low |

Cell entries: Y, yes; PY, partial yes; N, no; NA, not applicable (for example, no meta-analysis was undertaken). Critical domains are items 2, 4, 7, 9, 11, 13 and 15. Items: 1, population/PICO in question and criteria; 2, protocol established before the review (critical); 3, explanation of study-design selection; 4, comprehensive literature search (critical); 5, study selection in duplicate; 6, data extraction in duplicate; 7, list of excluded studies with justification (critical); 8, adequate description of included studies; 9, satisfactory risk-of-bias assessment (critical); 10, sources of funding of included studies; 11, appropriate statistical methods for synthesis (critical); 12, assessment of the effect of risk of bias on the synthesis; 13, risk of bias considered when interpreting results (critical); 14, explanation of heterogeneity; 15, assessment of publication bias (critical); 16, declaration of conflicts of interest. Overall confidence: High; Moderate; Low; C. low, critically low. Abbreviation: AMSTAR-2, A MeaSurement Tool to Assess systematic Reviews, version 2.

## Table S4. Certainty of the evidence: review-level detail (core-eligible reviews).

| **Review** | **Outcome** | **Comparator** | **Framework** | **Certainty (source)** | **Summary statement** |
| --- | --- | --- | --- | --- | --- |
| R06 Frade et al. | Fatigue | Placebo exercise | GRADE | Low  Reported by source review | MD −5.00 [95% CI −13.29, 3.29]; P = 0.24 |
| R06 Frade et al. | Withdrawals | Placebo exercise | GRADE | Very Low  Reported by source review | RR 2.73 [95% CI 0.34, 22.16] |
| R06 Frade et al. | Fatigue | Usual / standard care | GRADE | Low  Reported by source review | MD −0.59 [95% CI −1.40, 0.22]; I² = 0%; P = 0.15 |
| R06 Frade et al. | Disease activity | Usual / standard care | GRADE | Low  Reported by source review | MD −0.26 [95% CI −3.69, 3.17]; I²=90%; P = 0.88 |
| R06 Frade et al. | Withdrawals | Usual / standard care | GRADE | Very Low  Reported by source review | RR 0.92 [95% CI 0.53, 1.60]; I² = 0% |
| R06 Frade et al. | Fatigue | Active comparator | GRADE | Low  Reported by source review | SMD −0.51 [95% CI −0.88, −0.14]; I²=0%; P = 0.007 |
| R06 Frade et al. | Disease activity | Active comparator | GRADE | Moderate  Reported by source review | SMD 0.02 [95% CI −0.28, 0.32]; I² = 1%; P = 0.87 |
| R06 Frade et al. | Withdrawals | Active comparator | GRADE | Very Low  Reported by source review | RR 0.89 [95% CI 0.13, 5.94] |
| R09 O'Dwyer et al. | Disease activity | Usual / standard care | GRADE | Low  Assessed in this overview | Overview-applied Low certainty: downgraded for risk of bias and imprecision. MD 0.01 [95% CI −0.54 to 0.56]. |
| R09 O'Dwyer et al. | Fatigue | Usual / standard care | GRADE | Low  Assessed in this overview | Overview-applied Low certainty: downgraded for risk of bias and imprecision. MD −0.61 [95% CI −1.19 to −0.02]. |
| R16 Wu et al. | Fatigue | Usual / standard care | GRADE | Low  Assessed in this overview | Overview-applied Low certainty: downgraded for risk of bias and small-study imprecision. MD −0.52, 95% CI −0.91 to −0.13; P = 0.009. |
| R01 Alexanderson et al. | Fatigue | Usual / standard care | GRADE | Low  Reported by source review | Limited evidence supporting positive effect of aerobic exercise on fatigue. |
| R01 Alexanderson et al. | Disease activity | Usual / standard care | GRADE | Low  Reported by source review | Limited evidence that disease activity is unchanged by exercise. |
| R08 Gwinnutt et al. | Fatigue | Usual / standard care | Review’s own grading | Moderate  Reported by source review | Medium effect of aerobic exercise on fatigue in SLE. |
| R08 Gwinnutt et al. | Disease activity | Usual / standard care | Review’s own grading | Low  Reported by source review | Low-quality evidence that physical activity does not affect disease activity. |
| R12 Santos et al. | Fatigue | Usual / standard care | GRADE | Moderate a  Reported by source review | Physical activity or exercise reduced fatigue in SLE: SMD −0.54, 95% CI −1.07 to −0.01, P = 0.04. |
| R13 Silva-Ribeiro et al. | Disease activity | Reference / lower exposure | Ioannidis classification | Provisional Class IV Assessed in this overview | Physical activity: 6 studies, 9 effects, estimate −0.299 (0.089), 95% CI −0.473 to −0.124, converted r −0.290. The source review did not report all inputs required to verify Classes I–III; the classification is therefore provisional. |
| R13 Silva-Ribeiro et al. | Fatigue and pain | Reference / lower exposure | Ioannidis classification | Provisional Class IV Assessed in this overview | Physical activity: 9 studies, 19 effects, estimate −0.227, 95% CI −0.340 to −0.114. The source review did not report all inputs required to verify Classes I–III; the classification is therefore provisional. |

Certainty is shown for every review that reported or supported a certainty judgment, separated by evidence tier. GRADE was used for intervention comparisons. When a Core-eligible review did not report GRADE, two reviewers independently assessed the review-level estimate, starting randomized intervention evidence at high certainty and considering risk of bias, inconsistency, indirectness, imprecision and publication bias; disagreements were resolved by consensus. The Ioannidis hierarchy was applied provisionally to observational exposure associations because the source review did not report all inputs required to verify Classes I–III. The source column indicates whether a rating was reported by the source review or assessed in this overview. a Santos et al. reported Moderate certainty for this estimate; given the methodological limitations summarized in Table S3, a lower rating may be more appropriate. This review contributes to the evidence map only and does not inform the primary conclusions. Abbreviations: CI, confidence interval; GRADE, Grading of Recommendations Assessment, Development and Evaluation; I², heterogeneity statistic; MD, mean difference; SMD, standardized mean difference.

## Table S5. Corrected covered area overlap of primary studies between reviews.

| **Intervention / exposure cluster** | **Reviews, n** | **Primary studies, n (deduplicated)** | **CCA** | **Overlap level** | **Largest pairwise overlaps (shared studies)** |
| --- | --- | --- | --- | --- | --- |
| Structured exercise | 18 | 28 | 34.0% | Very high | R10–R14 (23); R03–R14 (19); R03–R10 (18) |
| Physical activity | 8 | 18 | 12.7% | High | R10–R14 (3); R03–R10 (3); R03–R14 (3) |
| Sedentary behavior | 4 | 7 | 14.3% | High | R10–R14 (1); R10–R13 (1); R13–R14 (1) |
| Lifestyle intervention (physical-activity component) | 2 | 2 | 100.0% | Very high | R10–R14 (2) |
| Overall (all clusters combined) | 18 | 55 | 19.6% | Very high | R10–R14 (29); R03–R14 (22); R03–R10 (21) |

The corrected covered area (CCA) quantifies how much the included reviews share the same primary studies within each prespecified cluster. It is calculated as (N − r) / (r × c − r), where N is the number of filled review-by-study cells, r is the number of unique primary studies and c is the number of reviews. Values are conventionally interpreted as slight (0–5%), moderate (6–10%), high (11–15%) or very high (>15%) overlap.

Largest pairwise overlaps list the review pairs sharing the most primary studies (number of shared studies in parentheses). The complete pairwise overlap matrix is shown in main manuscript Figure 4.

Implication. Overlap was high or very high in every cluster, so the reviews are not statistically independent; their estimates are therefore presented separately rather than pooled.

Abbreviations: CCA, corrected covered area; R01–R18, review identifiers as listed in main manuscript Table 1.

## Table S6. Primary studies and participants contributing to each pooled estimate reported by the Core-eligible reviews.

| **Pooled estimate (review; outcome; comparator)** | **Contributing primary studies (design as labelled by the source review; exercise n / comparator n)** | **Comparator used in the contributing trials** | **Outcome instrument(s)** | **Total participants, n** |
| --- | --- | --- | --- | --- |
| R09 O’Dwyer 2017; fatigue; usual / standard care. MD −0.61 (95% CI −1.19, −0.02); k = 2 | de Carvalho 2005 (quasi-randomized with matching; 41 / 19); Tench 2003 (randomized; 25 / 30) | Non-exercise control; no intervention | Fatigue Severity Scale | 115 |
| R16 Wu 2017; fatigue; usual / standard care. MD −0.52 (95% CI −0.91, −0.13); k = 3 | Ramsey-Goldman 2000 (randomized in this review, quasi-randomized in R09; 5 / 5); Tench 2003 (randomized; 33 / 32); de Carvalho 2005 (quasi-experimental; 41 / 19) | Non-exercise control; no intervention | Fatigue Severity Scale | 135 |
| R06 Frade 2023; fatigue; usual pharmacological care alone. MD −0.59 (95% CI −1.40, 0.22); k = 2 | Avaux 2016 (quasi-randomized; 31 / 8, two exercise arms combined); Tench 2003 (randomized; 33 / 32) | Usual pharmacological care alone | Fatigue Severity Scale | 104 |
| R06 Frade 2023; fatigue; placebo exercise. MD −5.00 (95% CI −13.29, 3.29); k = 1 | Lopes-Souza 2021 (randomized; 8 / 9) | Placebo exercise plus usual care | FACIT-Fatigue | 17 |
| R06 Frade 2023; fatigue; active comparator. SMD −0.51 (95% CI −0.88, −0.14); k = 2 | Keramiotou 2020 (randomized; 28 / 30); Tench 2003 (randomized; 33 / 28) | Joint aids plus disease information; relaxation therapy | LupusQoL fatigue domain; Fatigue Severity Scale | 119 |
| R16 Wu 2017; SF-36 vitality; usual / standard care. MD 14.98 (95% CI 7.45, 22.52); k = 2 | de Carvalho 2005; Tench 2003 (arm sizes as above) | Non-exercise control; no intervention | SF-36 vitality domain | Not stated separately in the source review |
| R09 O’Dwyer 2017; disease activity; usual / standard care. MD 0.01 (95% CI −0.54, 0.56); k = 3 | dos Reis-Neto 2013 (quasi-randomized; 18 / 20); Miossi 2012 (randomized; 15 / 13); Abrahão 2016 (randomized; 42 / 19) | Non-exercise control | SLEDAI | 127 |
| R06 Frade 2023; disease activity; usual pharmacological care alone. MD −0.26 (95% CI −3.69, 3.17); k = 2 | Boström 2016 (randomized; 18 / 17); Tench 2003 (randomized; 33 / 32) | Usual pharmacological care alone | SLEDAI; SLAM | 100 |
| R06 Frade 2023; disease activity; active comparator. SMD 0.02 (95% CI −0.28, 0.32); k = 4 | Abrahão 2016 (42 / 19); dos Reis-Neto 2013 (18 / 20); Miossi 2012 (14 / 10); Tench 2003 (33 / 28) | Disease information; relaxation therapy | SLEDAI; SLAM | 184 |
| R06 Frade 2023; withdrawals for any reason; usual pharmacological care alone. RR 0.92 (95% CI 0.53, 1.60); k = 6 | Avaux 2016 (33 / 9); Benatti 2015 (20 / 20); Benatti 2018 (14 / 15); Boström 2016 (18 / 17); Hashemi 2022 (14 / 10); Tench 2003 (33 / 32) | Usual pharmacological care alone | Withdrawals for any reason | 235 |
| R06 Frade 2023; withdrawals for any reason; placebo exercise. RR 2.73 (95% CI 0.34, 22.16); k = 1 | Lopes-Souza 2021 (11 / 10) | Placebo exercise plus usual care | Withdrawals for any reason | 21 |
| R06 Frade 2023; withdrawals for any reason; active comparator. RR 0.89 (95% CI 0.13, 5.94); k = 7 | Abrahão 2016 (42 / 21); Daltroy 1995 (16 / 18); dos Reis-Neto 2013 (23 / 21); Kao 2021 (12 / 11); Keramiotou 2020 (32 / 30); Miossi 2012 (15 / 15); Tench 2003 (33 / 28) | Disease information; relaxation therapy | Withdrawals for any reason | 317 |

Numbers are those used for the analysis in the source review and were verified against that review’s forest plots or summary tables. Arm-level counts may represent analyzed participants rather than all participants originally randomized and may differ across reviews because of attrition, arm selection or inconsistent extraction. Reviews are identified by the codes used in main manuscript Table 1.

Two features are relevant to interpretation. First, every pooled fatigue estimate for exercise versus usual or standard care derives from an overlapping pool of four primary trials — Tench et al. 2003, de Carvalho et al. 2005, Ramsey-Goldman et al. 2000 and Avaux et al. 2016. Counting each trial once and using the fullest relevant arm-level count reported across source reviews, these represent 174 participants in the analyzed arms; no single pooled estimate includes more than 135 participants. Second, the source reviews did not classify or extract every trial identically. The arm sizes recorded for Tench et al. 2003 differ between reviews, and de Carvalho et al. 2005 and Ramsey-Goldman et al. 2000 are labelled randomized by some reviews and quasi-randomized or quasi-experimental by others. This table preserves the label and count used by each source review rather than imposing a retrospective reclassification.

Abbreviations: CI, confidence interval; FACIT, Functional Assessment of Chronic Illness Therapy; k, number of contributing primary studies; MD, mean difference; RR, risk ratio; SF-36, 36-item Short-Form Health Survey; SLAM, Systemic Lupus Activity Measure; SLEDAI, Systemic Lupus Erythematosus Disease Activity Index; SMD, standardized mean difference.

## Table S7. Sensitivity synthesis of the Core-eligible evidence restricted by AMSTAR-2 overall confidence.

| **Outcome and comparator** | **Primary synthesis (all seven Core-eligible reviews)** | **Restricted to high or low confidence (R02, R06, R09, R15)** | **Restricted to high confidence only (R06)** |
| --- | --- | --- | --- |
| Fatigue; usual / standard care | Three pooled estimates; two significant (R09, R16), one not (R06); GRADE low | Two pooled estimates; R09 significant, R06 not; the significant estimate from R16 (critically low confidence) no longer contributes | One estimate (R06); no statistically significant difference |
| Fatigue; placebo exercise | One estimate (R06); not significant; GRADE low | Unchanged | Unchanged |
| Fatigue; active comparator | One estimate (R06); significant reduction; GRADE low | Unchanged | Unchanged |
| SF-36 vitality; usual / standard care | One estimate (R16); favors exercise | No estimate remains | No estimate remains |
| Disease activity; usual / standard care | Two estimates (R06, R09); neither significant; GRADE low | Unchanged | One estimate (R06); not significant |
| Disease activity; active comparator | One estimate (R06); not significant; GRADE moderate | Unchanged | Unchanged |
| Withdrawals for any reason (all comparators) | Three estimates (R06); none significant; GRADE very low | Unchanged | Unchanged |
| Serious adverse events and disease flares | Narrative only across R02, R03, R06, R15, R16 and R18; not graded; no consistent signal, but safety not established | Narrative only across R02, R06 and R15; not graded; safety not established | Narrative only (R06); not graded; safety not established |

AMSTAR-2 overall confidence among the seven Core-eligible reviews was high for R06 (Frade et al. 2023), low for R02 (Ayán et al. 2018), R09 (O’Dwyer et al. 2017) and R15 (Vandenbulcke et al. 2023), and critically low for R03 (Blaess et al. 2023), R16 (Wu et al. 2017) and R18 (Zouganeli et al. 2025). Core eligibility was defined by direct relevance to the prespecified PICO and design criteria and is therefore independent of methodological quality.

The restricted set therefore comprises R02, R06, R09 and R15, and excludes R03, R16 and R18. The restricted syntheses are descriptive: no estimate was re-pooled, and only the set of contributing reviews changes. Direction of effect for disease activity and for withdrawals is unchanged in both restricted sets; the fatigue evidence becomes more conservative, because one of the two statistically significant fatigue estimates versus usual care comes from a review of critically low confidence.

Abbreviations: AMSTAR-2, A MeaSurement Tool to Assess systematic Reviews, version 2; GRADE, Grading of Recommendations Assessment, Development and Evaluation; SF-36, 36-item Short-Form Health Survey.

## Table S8. Complete containment (nesting) of primary-study sets among the included reviews.

| **Review** | **Primary studies contributed, n** | **AMSTAR-2 confidence** | **Primary-study set fully contained within** | **Primary studies unique to this review, n** |
| --- | --- | --- | --- | --- |
| R01 Alexanderson and Boström 2020 | 8 | Critically low | R02; R03; R09 | 0 |
| R02 Ayán et al. 2018 | 12 | Low | None | 0 |
| R03 Blaess et al. 2023 | 37 | Critically low | None | 11 |
| R04 Del Pino-Sedeño et al. 2016 | 5 | Critically low | R03; R11; R17 | 0 |
| R05 Fangtham et al. 2019 | 7 | Critically low | R01; R02; R03; R08; R09 | 0 |
| R06 Frade et al. 2023 | 12 | High | None | 0 |
| R07 Geertsema-Hoeve et al. 2024 | 4 | Critically low | R10; R14; R15; R18 | 0 |
| R08 Gwinnutt et al. 2022 | 11 | Critically low | None | 0 |
| R09 O’Dwyer et al. 2017 | 10 | Low | R02 | 0 |
| R10 Parodis et al. 2023 | 29 | Critically low | R14 | 0 |
| R11 Rodríguez Huerta et al. 2016 | 9 | Critically low | None | 0 |
| R12 Santos et al. 2023 | 9 | Low | R15 | 0 |
| R13 Silva-Ribeiro et al. 2025 | 14 | Low | None | 5 |
| R14 Tsoi et al. 2024 | 33 | Critically low | None | 3 |
| R15 Vandenbulcke et al. 2023 | 17 | Low | None | 0 |
| R16 Wu et al. 2017 | 3 | Critically low | R02; R03; R04; R08; R09; R10; R11; R14; R15; R17 | 0 |
| R17 Yuen and Cunningham 2014 | 7 | Critically low | None | 0 |
| R18 Zouganeli et al. 2025 | 11 | Critically low | None | 0 |

A review is recorded as fully contained within another when every primary study it includes is also included in that other review, and the other review includes at least one further study. Containment was computed from the deduplicated study-by-review matrix used for the corrected covered area analysis (Table S5), after linked reports of the same protocol were collapsed into a single primary study.

Eight of the eighteen reviews (R01, R04, R05, R07, R09, R10, R12 and R16) add no primary study that is not already contained in a larger review, and only three reviews (R03, R13 and R14) contribute at least one primary study that no other included review contains. Nineteen of the 55 deduplicated primary studies were included by a single review. Containment is asymmetric and is not visible in the pairwise overlap matrix shown in main manuscript Figure 4; it is displayed graphically in Supplementary Figure S1.

Abbreviations: AMSTAR-2, A MeaSurement Tool to Assess systematic Reviews, version 2; R01–R18, review identifiers as listed in main manuscript Table 1.

## Supplementary Figure S1. Containment hierarchy of primary-study sets among the included reviews.

Each node is an included review, labelled with its identifier and the number of primary studies it contributed. An arrow runs from a review to a larger review that contains its entire primary-study set. Reviews with no outgoing arrow are not fully contained within any other review. Node fill indicates AMSTAR-2 overall confidence. The figure is supplied as a separate TIFF file. Abbreviations: AMSTAR-2, A MeaSurement Tool to Assess systematic Reviews, version 2; R01–R18, review identifiers as listed in main manuscript Table 1.
